# A Dispersive Migration in the Atlantic Puffin and Its Implications for Migratory Navigation

**DOI:** 10.1371/journal.pone.0021336

**Published:** 2011-07-20

**Authors:** Tim Guilford, Robin Freeman, Dave Boyle, Ben Dean, Holly Kirk, Richard Phillips, Chris Perrins

**Affiliations:** 1 Department of Zoology, University of Oxford, Oxford, Oxfordshire, United Kingdom; 2 Computational Ecology and Environmental Science, Microsoft Research Cambridge, Cambridge, Cambridgeshire, United Kingdom; 3 Edward Grey Institute of Field Ornithology, University of Oxford, Oxford, Oxfordshire, United Kingdom; 4 British Antarctic Survey, Natural Environment Research Council, Cambridge, Cambridgeshire, United Kingdom; Institut Pluridisciplinaire Hubert Curien, France

## Abstract

Navigational control of avian migration is understood, largely from the study of terrestrial birds, to depend on either genetically or culturally inherited information. By tracking the individual migrations of Atlantic Puffins, *Fratercula arctica*, in successive years using geolocators, we describe migratory behaviour in a pelagic seabird that is apparently incompatible with this view. Puffins do not migrate to a single overwintering area, but follow a dispersive pattern of movements changing through the non-breeding period, showing great variability in travel distances and directions. Despite this within-population variability, individuals show remarkable consistency in their own migratory routes among years. This combination of complex population dispersion and individual route fidelity cannot easily be accounted for in terms of genetic inheritance of compass instructions, or cultural inheritance of traditional routes. We suggest that a mechanism of individual exploration and acquired navigational memory may provide the dominant control over Puffin migration, and potentially some other pelagic seabirds, despite the apparently featureless nature of the ocean.

## Introduction

Masters of migration, birds have formed the core model for our understanding of animal migration for decades [1], leading to an orthodoxy that migratory patterns are dominated either by genetically inherited compass information, or culturally inherited routes. Whilst the navigational capacities of terrestrial birds have been more intensely studied than those of any other animal group, following the behaviour of individuals during natural migrations has always proved difficult, leaving unanswered important questions about how individuals actually control navigation over very long distances [2]. The latter problem is especially acute for seabirds, such as Atlantic Puffins, *Fratercula arctica*, that migrate over open ocean, where available cues may differ from those over land [3].

Puffins breed on relatively isolated cliff slopes and islands around the North Atlantic in the northern summer, pursuit diving for pelagic fish in local waters, then leave their dense colonies in late summer on migration. The overwintering destinations of Puffins (in common with many pelagic seabirds) have never been pin-pointed clearly from recoveries of ringed birds that have died, suggesting that they spend most of the time in the open ocean (recently confirmed by Harris *et al.* [4]) probably distributed widely around the North Atlantic, North Sea, and even the Mediterranean (for UK breeders) [5]. There is little evidence of concentrated overwintering areas, and relatively few, scattered winter sightings at sea, suggesting that migratory routes may be highly variable. Furthermore, fledglings leave at night, apparently alone, long before adults normally abandon the colony for the winter [5], so they cannot be following their parents. Together these facts suggest that navigational control of migration in the Puffin may not conform well to the established theories of genetic or cultural inheritance. The aim of this study, therefore, was to characterize the consistency of migratory behaviour, both between and within individuals, by tracking them with miniature archival light loggers (geolocators). In particular we aimed to determine whether an individual's migratory route was consistent from one year to the next, since this would indicate that an individual's decisions involving navigational information were capable of remaining consistent over very long time periods even if individual destinations were variable.

## Materials and Methods

Ethics Statement: All work was conducted after ethical approval by the Countryside Council for Wales (Licence numbers: OTH/SB/04/2007; OTH/SB/03/2008; OTH:SB-04-2009), the Skomer Island Advisory Committee (no licence or permits are required or issued), and the British Trust for Ornithology's Unconventional Methods Committee (BTO permits: Guilford, 5311; Perrins, 660).

We tracked birds using geolocators (Mk 13 or Mk14; British Antarctic Survey, Cambridge) at a compact sub-colony of Puffins at The Isthmus on Skomer Island, Wales, UK, (51 degrees 44′N; 5 degrees 19′W) where breeding success has been monitored for many years [6], [7]. Stored light level measurements are used to derive dawn and dusk transition times from which it is possible to estimate approximate location anywhere on earth except during periods of a few weeks around the equinoxes (see reference [8] for analysis of position accuracy). Birds were caught on entry to, or exit from, their breeding burrow during late chick rearing using purse nets under constant surveillance and taken to a nearby laboratory for weighing, ringing, and geolocator attachment using a Darvic leg ring (which usually took less than 12 minutes). Tagged birds were re-caught during the next or a subsequent breeding season in the same way, and data downloaded from the device *in situ*, or the device removed. The mass of device and attachment was approximately 2 g, comprising a maximum load of 0.6% body mass for birds of minimum mass 350 g. In 2007 we conducted a pilot study of just 6 individuals in order to check for potential deleterious effects. All birds returned to the colony the following year. On this basis we expanded the study, with 18 devices deployed in 2008 (13 new birds and 5 birds that had been tracked the previous year), and 26 in 2009 (8 new birds, and 18 previously tracked birds). In total, the deployments amounted to 27 birds over 49 bird-years. Details of the fates of birds and their deployed geolocators are given in Table S1.

Geolocator light data were decompressed using BASTrack and then processed using TransEdit and BirdTracker software (BAS) and Matlab (Mathworks, Natick, Mass.), using a light threshold of 10 and an elevation angle that most closely matched estimated locations during ground truthing periods to the known colony position (usually −4.5). Sets of valid locations for each tracked individual were extracted from the raw data in the following way. Locations based on unclear day/night transitions in light curves were identified during processing and removed, as were those resulting from unrealistically short dark periods (<4 hours), or proximity to the spring and autumn equinoxes. Remaining locations were further filtered to remove unrealistic movements (more than 2 degrees in 24 hours). Only months outside the breeding season (August-March) were included in analyses: 1), because we were interested in the migration period; and 2), because timing of dawn and dusk is unavailable for birds in burrows.

**Figure 1:**
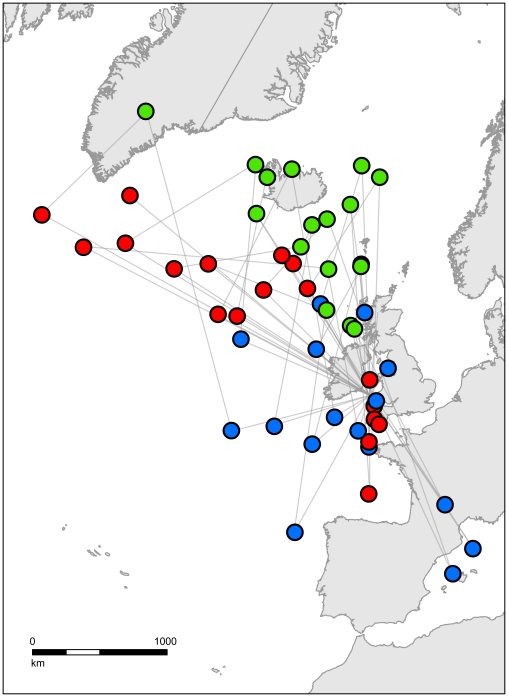
Dispersive migration in the Atlantic Puffin. Patterns of migratory movements for 18 Puffins tracked using geolocators are shown as median individual position estimates during three months outside the breeding season: August (red); October (green); and February (blue). Lines join each individual bird's successive positions, but do not indicate the path travelled.

The error inherent in light-based geolocation tracking is often too great [8] to provide accurate daily movements. We visualized the overall movement patterns using monthly spatial medians of the valid daily latitude and longitude estimates to provide each bird's approximate position during three selected months spaced across the whole migration period. To gauge the levels of combined movement and error within each month the reader is referred to the raw plots of validated locations in figures S1, S2, S3, S4, S5, S6, S7, S8, S9, S10, S11, S12, S13, S14, S15, S16, S17, S18, S19, S20, S21, S22, S23, S24, S25, S26, presented separately for every bird, which also show the monthly positions not included in Figure 1 summary visualization. However, to determine the consistency of migratory routes either between or within individuals required a more sophisticated analysis. We used a novel technique, based on nearest neighbour analysis, which we recently developed for analyzing route-fidelity in the GPS tracks of flying pigeons [9], and which quantifies the spatial similarity between a focal track and a comparison track. For each valid location on the focal track, the distance to its nearest neighbour on the comparison track is calculated within a window of 15 days either side (to minimize the chance that apparent dissimilarity may be generated by timing differences of otherwise similar routes). The mean of these nearest-neighbour distances across the whole track provides a metric of track similarity. The critical question concerns whether individuals are faithful to their own routes between years relative to the routes taken by others. To assess this “route fidelity” we used randomization tests to construct a null distribution of route similarity amongst pairs of routes drawn from the overall set of single migrations. Significance was assessed by calculating how rarely the differences amongst a randomly drawn set of route pairs were as extreme (two-tailed) or as small (one-tailed) as those displayed by the set individuals' own route pairs.

## Results

26 of the 27 birds were seen back at the colony in the year following deployment or subsequently, indicating that survival over the winter immediately following deployment was at least 96%. Over a total of 49 bird-deployment-years, in only three cases has a bird not been subsequently sighted suggesting that it may not have survived the winter, indicating a minimum annual survival rate of 94% (average survival rates at Skomer are usually high: 95% between 1972 and 1977 [6]; 93% between 1984 and 2001 [7]; 84% in 2007/8, and 92% in 2008/9). No immediate nest desertions were observed. 38 out of 44 breeding birds for which the fate was known were successful (86% fledging success). This level of success was comparable to that in the colony as a whole over this three year period (67% in 2008, 77% in 2009, 80% in 2010, Boyle, D. Personal Communication). Geolocator deployments therefore appeared to cause no measurable impact.

Migration data were obtained from 18 of the 27 study birds in at least one year (missing data were due to device failure or inability to recapture birds, detailed in Table S1). The first or most complete migration recorded for each individual, summarized for clarity as median positions for August, October, and February, is plotted in Fig. 1 (complete plots of all valid winter locations for each individual are provided in figures S1, S2, S3, S4, S5, S6, S7, S8, S9, S10, S11, S12, S13, S14, S15, S16, S17, S18, S19, S20, S21, S22, S23, S24, S25, S26). Tracked birds showed a complex pattern of migratory movements during the non-breeding months, starting with highly dispersive movements into the Atlantic. In August most birds migrate away from the colony, most in a NW-W direction, some as far as Greenland, some more locally, whilst some move southwards towards France and Biscay. In autumn they move northwards or north eastwards into the North Atlantic, and then later in the winter they move southwards, some as far as the Mediterranean, before returning (from a variety of directions) to the colony in spring.

**Figure 2:**
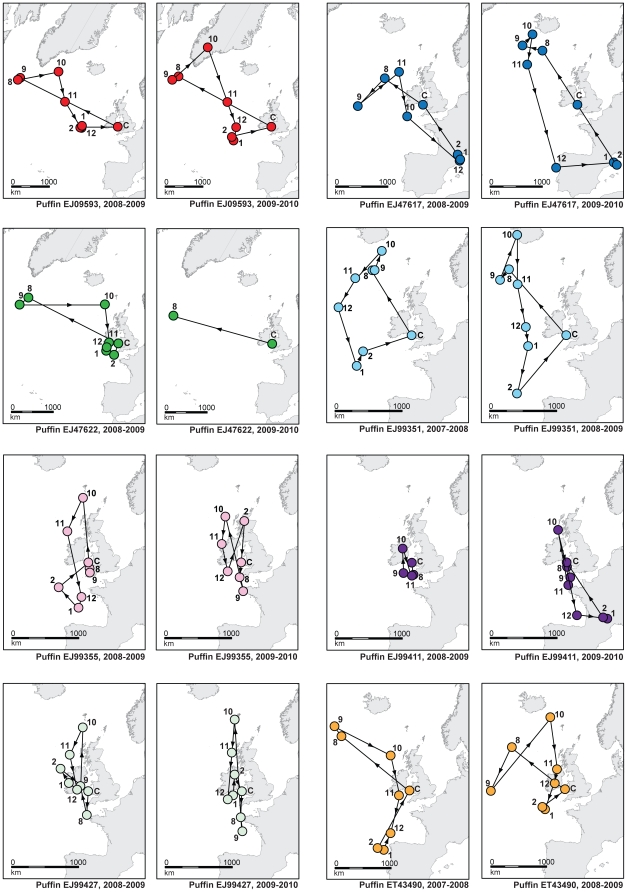
Migratory tracks of 8 individual Puffins in two successive years. Each individual is indicated in a different colour and position estimates are given as monthly medians of available data, with the month indicated by a number (January = 1). Breeding season data are excluded, and the colony location marked by C. Lines join each individual bird's successive positions, but do not indicate the path travelled.

Furthermore, our data show that despite the population variability these are well directed migrations and not simple dispersal movements. The monthly median positions for eight birds tracked for two successive migrations (partial data in two cases) are plotted in figure 2. In one case (EJ99355) data were used from 2007/8 and 2009/10, with partial data from the intervening year left unused. In every case the individual's route is conserved, despite the idiosyncratic nature of the bird's movements. Most dramatically, whilst one bird migrates north west each year to the western Atlantic, another migrates south east each year to the central Mediterranean after first spending the autumn south of Iceland.

**Figure 3:**
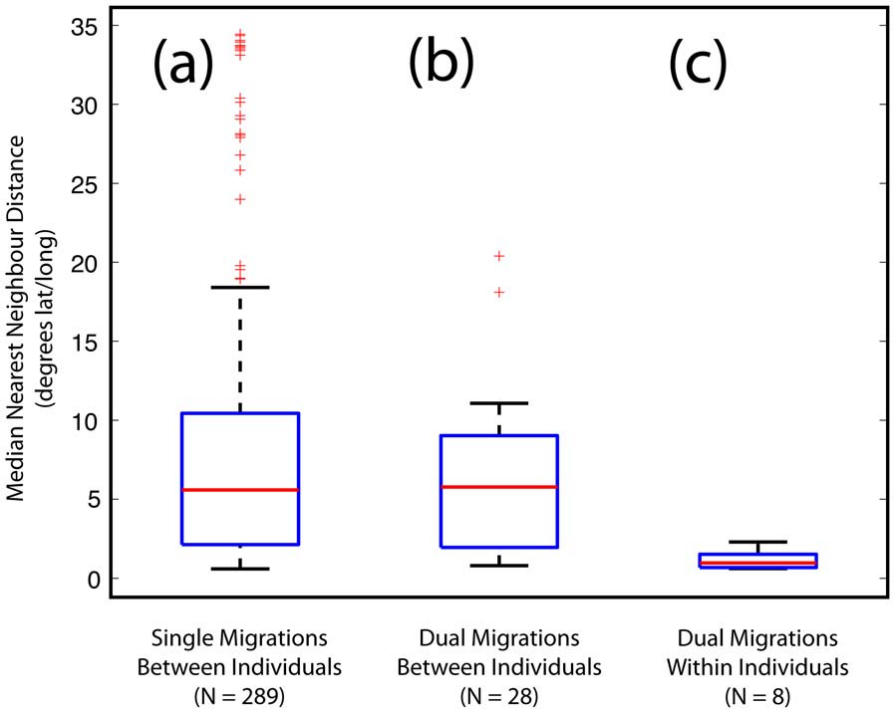
Migration routes are more similar within than between individuals. The Box-plots show average nearest neighbour distances between points within a moving 30 day window along pairs of migratory tracks. Left (a) includes all possible pairs of tracks from birds for whom we have just a single migration recorded. Centre (b) includes distances between pairs of tracks from different birds for whom we have two migrations recorded. Right (c) is the set of distances between pairs of tracks completed by the same individuals.

Figure 3 shows the distribution of median nearest neighbour distances: (a), between all possible pairings of birds with just a single recorded route (N = 289); (b), between all possible pairs of routes of birds with two recorded migrations but excluding each individual's own subsequent route (N = 28); and (c), between each individual's own two recorded migrations (N = 8). The difference of the distances between group (a) and group (c) was compared to the difference between randomly generated groups of the same size drawn from all possible route pairings. In 100 000 randomisations we found very few cases in which this value was as large as that observed between (a) and (c)(p<0.018, two-tailed), and no cases where the randomly generated pairs were as similar as in the set of dual migrations (c) (p≈0, one-tailed). In contrast, there was very little difference between the distances of groups (a) and (b), indicating that the routes taken by dual migration birds were as diverse as those taken by the tracked birds as a whole.

## Discussion

Our data show that Puffins from Skomer have both a complex and highly dispersive migration, a finding consistent with recent results for conspecifics breeding in north east Britain [4]. Nonetheless, individuals are significantly more likely to follow a migration similar to their own previous route than that of another Puffin. Nearest neighbour analysis demonstrates that despite the relatively small sample and the inherent noisiness of geolocator data, this combination of dispersive migration and route fidelity leads to individually idiosyncratic behaviour that is statistically robust. Whilst much larger and longer studies would be required to understand what *controls* variation between birds, or between years, it is nonetheless clear that our finding cannot be an anomaly due to the sample of birds that we tracked on multiple migration: dual-tracked birds are statistically just as a diverse in their migration routes as are the larger sample of singly tracked birds. What kind of navigational control can account for this combination of population dispersion and individual route fidelity?

Current understanding of the control of first migration suggests either 1) that approximate movement direction, and distance, are coded for genetically (so-called ‘clock and compass’), or, 2), that traditional routes are learnt from parents or other conspecifics during social migration [10], [11], [12]. Subsequent migrations may be fixed by the learning of key landscape (or seascape) features, or honed by acquired experience of navigational map factors *en-route* (possibly magnetic [13]–[15] or olfactory [3], [16]). Whilst individual experience can lead to idiosyncrasies in the details of individual routes, the core pattern is inherited either genetically or culturally. This current understanding accounts poorly for Puffin migration.

Our results show a complex pattern which is both multi-directional and changing through the non-breeding season. It is possible that such behaviour may be under genetic control, conforming to current theory, but this seems unlikely. Genetically pre-programmed changes in orientation behaviour during the migratory period have been found in some passerines apparently providing appropriate control of their curved migratory routes [17], and sometimes such changes may be triggered by appropriate regional cues [1], [18]. However, the complex sequence of directional movements identified here would require considerably more complexity in genetic control than has hitherto been discovered for birds.

Nevertheless, the more striking finding is the Puffins' individual diversity of migratory movements, and this would also require an unusual system of genetic control since there is limited consistency between individuals. Where distinctly different migratory routes have been studied in detail there is often a migratory divide, with characteristic genetically encoded orientation tendencies in populations either side [1], [10]. We do not yet know whether there is any genetic orientation tendency in Puffins, but their extremely close breeding proximity in our study shows that there is no migratory divide. Sympatric genetic polymorphism in directional tendency is possible, as is some kind of conditional switch, but neither has been investigated yet in any migratory species.

The alternative, that routes are culturally inherited, also seems unlikely because Puffins are relatively unsocial away from the colony outside the breeding season [5]. Off-shore surveys around Britain indicate that between September and February the majority of sightings involve solitary birds (81% of sighting events, 63% of birds sighted), with pairs or very small groups observed infrequently. During August, aggregations are more common (70% of sighting events solitary, 39% of birds), suggesting that opportunities for following or joining conspecifics may in fact vary through the migratory period (Webb, A., Unpublished Observations. See [19] for methods and extent of data collection). More important, however, the young fledge at night without their parents, apparently disappearing at least beyond sight of the colony [5], making parental transmission of routes extremely unlikely. Furthermore, most fledging occurs before adults generally leave the colony on migration, so there is little opportunity for systematic transmission of route information from adult to young. Occasional or haphazard following of other birds (conspecifics or otherwise) remains a possibility, but systematic cultural inheritance of migratory routes is unlikely.

In common with many pelagic seabirds Puffins can stop anywhere at sea, and feed if resources are available. Furthermore, suitable overwintering habitat may be both spatially diverse and relatively dynamic. These factors may favour selection of less rigid migratory control, and allow more extensive exploratory movements. One possible hypothesis therefore, which we call the “exploration-refinement hypothesis”, is that Puffins have neither strict genetic nor cultural control of migration, but instead rely on a system of large-scale exploratory movements during the pre-breeding years which become refined into an individual migratory route and set of destinations through learning. For Puffins, as for most seabirds, we are at a very early stage in understanding the precise mechanisms of orientation, but studying how patterns of movement develop between fledging and first return to the colony, and investigating heritability of migration routes, timing and destinations would be important first steps in determining how widespread individually learnt migration control is, and what navigational systems might be involved.

The inadequacy of established hypotheses to account for migratory orientation control in pelagic seabirds, and the likelihood of a greater role for experience, has been hinted at before, prompted by individual consistencies in the long distance movements of albatrosses [1], [20], [21]. Fidelity even for distant foraging areas located over shelf edges can be remarkably high [22], suggesting similarities with the learnt migratory movements implied here. Route-fidelity has also been noted in other groups of oceanic migrants, such as Northern Elephant Seals *Mirounga angustirostris* [23], although not yet in combination with complex dispersive migration. Paradoxically (because our evidence for it is individual route fidelity) learning may also help account for apparent lack of fidelity in overwintering sites [24] if individuals can learn to switch strategically between multiple destinations and routes. Evidence for a greater role for learning may in fact be quite widespread even though its role in shaping migration strategies has not been widely appreciated or formally developed. Especially in the light of emerging modern tracking technologies we believe it is now time to consider explicitly whether the “exploration-refinement” hypothesis could help explain observed movement patterns in future studies of vertebrate migration.

## Supporting Information

Figure S1 **Filtered (valid) geolocator position estimates (small circles), and monthly spatial median positions (large circles) for Puffin EJ99351, colour coded by month during the 2007–2008 non-breeding season.**
(TIFF)

Figure S2 **Filtered (valid) geolocator position estimates (small circles), and monthly spatial median positions (large circles) for Puffin EJ99352, colour coded by month during the 2007–2008 non-breeding season.**
(TIFF)

Figure S3 **Filtered (valid) geolocator position estimates (small circles), and monthly spatial median positions (large circles) for Puffin EJ99354, colour coded by month during the 2007–2008 non-breeding season.**
(TIFF)

Figure S4 **Filtered (valid) geolocator position estimates (small circles), and monthly spatial median positions (large circles) for Puffin EJ99355, colour coded by month during the 2007–2008 non-breeding season.**
(TIFF)

Figure S5 **Filtered (valid) geolocator position estimates (small circles), and monthly spatial median positions (large circles) for Puffin ET43490, colour coded by month during the 2007–2008 non-breeding season.**
(TIFF)

Figure S6 **Filtered (valid) geolocator position estimates (small circles), and monthly spatial median positions (large circles) for Puffin EJ09593, colour coded by month during the 2008–2009 non-breeding season.**
(TIFF)

Figure S7 **Filtered (valid) geolocator position estimates (small circles), and monthly spatial median positions (large circles) for Puffin EJ47617, colour coded by month during the 2008–2009 non-breeding season.**
(TIFF)

Figure S8 **Filtered (valid) geolocator position estimates (small circles), and monthly spatial median positions (large circles) for Puffin EJ47622, colour coded by month during the 2008–2009 non-breeding season.**
(TIFF)

Figure S9 **Filtered (valid) geolocator position estimates (small circles), and monthly spatial median positions (large circles) for Puffin EJ47623, colour coded by month during the 2008–2009 non-breeding season.**
(TIFF)

Figure S10 **Filtered (valid) geolocator position estimates (small circles), and monthly spatial median positions (large circles) for Puffin EJ47624, colour coded by month during the 2008–2009 non-breeding season.**
(TIFF)

Figure S11 **Filtered (valid) geolocator position estimates (small circles), and monthly spatial median positions (large circles) for Puffin EJ99351, colour coded by month during the 2008–2009 non-breeding season.**
(TIFF)

Figure S12 **Filtered (valid) geolocator position estimates (small circles), and monthly spatial median positions (large circles) for Puffin EJ99411, colour coded by month during the 2008–2009 non-breeding season.**
(TIFF)

Figure S13 **Filtered (valid) geolocator position estimates (small circles), and monthly spatial median positions (large circles) for Puffin EJ99424, colour coded by month during the 2008–2009 non-breeding season.**
(TIFF)

Figure S14 **Filtered (valid) geolocator position estimates (small circles), and monthly spatial median positions (large circles) for Puffin EJ99427, colour coded by month during the 2008–2009 non-breeding season.**
(TIFF)

Figure S15 **Filtered (valid) geolocator position estimates (small circles), and monthly spatial median positions (large circles) for Puffin EJ43490, colour coded by month during the 2008–2009 non-breeding season.**
(TIFF)

Figure S16 **Filtered (valid) geolocator position estimates (small circles), and monthly spatial median positions (large circles) for Puffin EJ09593, colour coded by month during the 2009–2010 non-breeding season.**
(TIFF)

Figure S17 **Filtered (valid) geolocator position estimates (small circles), and monthly spatial median positions (large circles) for Puffin EJ47617, colour coded by month during the 2009–2010 non-breeding season.**
(TIFF)

Figure S18 **Filtered (valid) geolocator position estimates (small circles), and monthly spatial median positions (large circles) for Puffin EJ47622, colour coded by month during the 2009–2010 non-breeding season.**
(TIFF)

Figure S19 **Filtered (valid) geolocator position estimates (small circles), and monthly spatial median positions (large circles) for Puffin EJ47625, colour coded by month during the 2009–2010 non-breeding season.**
(TIFF)

Figure S20 **Filtered (valid) geolocator position estimates (small circles), and monthly spatial median positions (large circles) for Puffin EJ60575, colour coded by month during the 2009–2010 non-breeding season.**
(TIFF)

Figure S21 **Filtered (valid) geolocator position estimates (small circles), and monthly spatial median positions (large circles) for Puffin EJ99355, colour coded by month during the 2009–2010 non-breeding season.**
(TIFF)

Figure S22 **Filtered (valid) geolocator position estimates (small circles), and monthly spatial median positions (large circles) for Puffin EJ99411, colour coded by month during the 2009–2010 non-breeding season.**
(TIFF)

Figure S23 **Filtered (valid) geolocator position estimates (small circles), and monthly spatial median positions (large circles) for Puffin EJ99427, colour coded by month during the 2009–2010 non-breeding season.**
(TIFF)

Figure S24 **Filtered (valid) geolocator position estimates (small circles), and monthly spatial median positions (large circles) for Puffin EL60571, colour coded by month during the 2009–2010 non-breeding season.**
(TIFF)

Figure S25 **Filtered (valid) geolocator position estimates (small circles), and monthly spatial median positions (large circles) for Puffin EL60579, colour coded by month during the 2009–2010 non-breeding season.**
(TIFF)

Figure S26 **Filtered (valid) geolocator position estimates (small circles), and monthly spatial median positions (large circles) for Puffin EL60648, colour coded by month during the 2009–2010 non-breeding season.**
(TIFF)

Table S1 **Details of each individual Puffin used in the study including geolocator deployment periods, recaptures, the fate of geolocator data, the supplementary figure numbers containing displaying these data, and the breeding success of the bird in the season following deployment.**
(DOC)
