# Supplementary figures and images for: A Dispersive Migration in the Atlantic Puffin and Its Implications for Migratory Navigation
Source: PLoS One. 2011 Jul 20;6(7):e21336. doi: 10.1371/journal.pone.0021336 (PMC3140476; doi:10.1371/journal.pone.0021336)

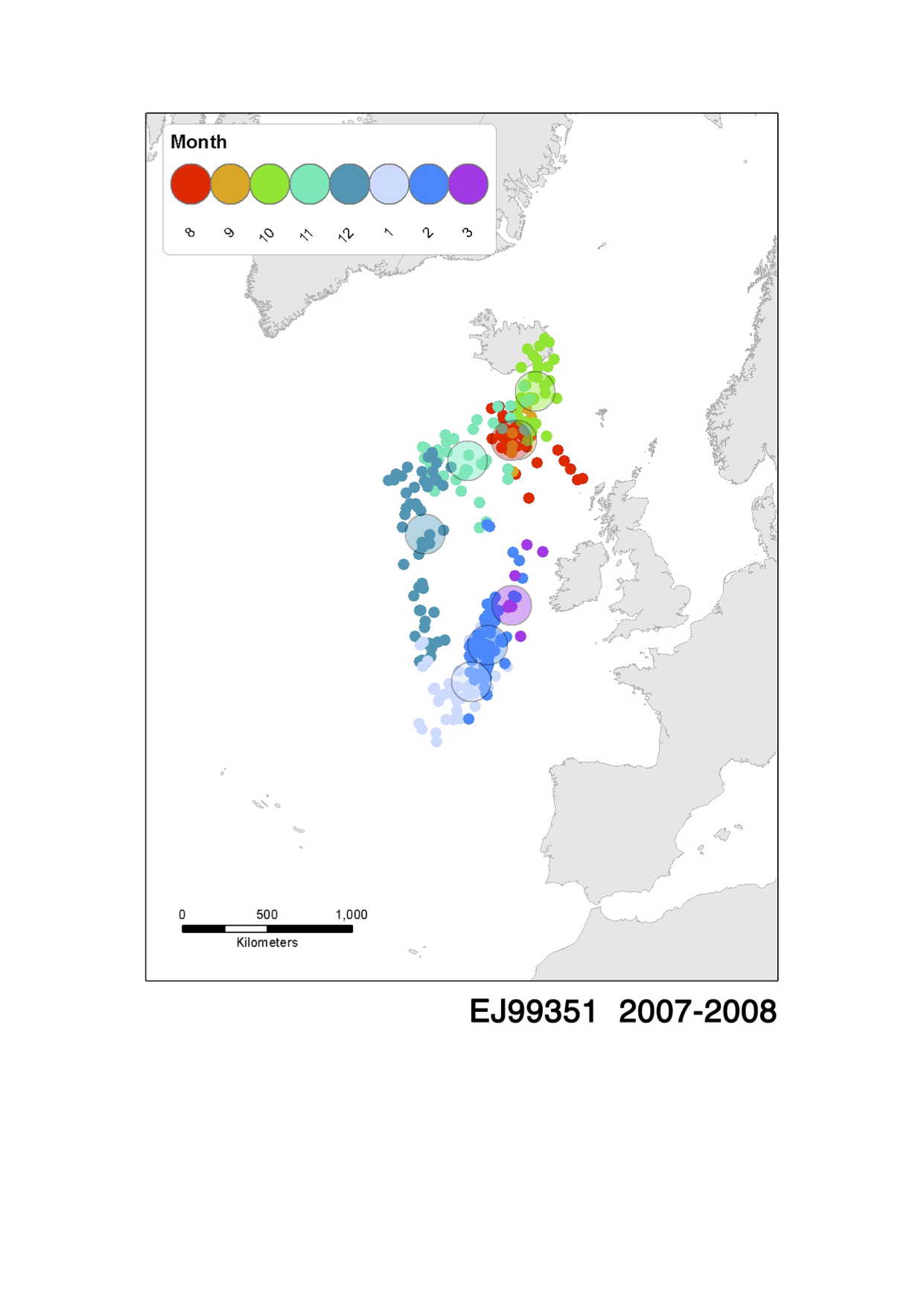

Supplement: Figure S1 — Filtered (valid) geolocator position estimates (small circles), and monthly spatial median positions (large circles) for Puffin EJ99351, colour coded by month during the 2007–2008 non-breeding season. (TIFF) [file pone.0021336.s001.tiff]

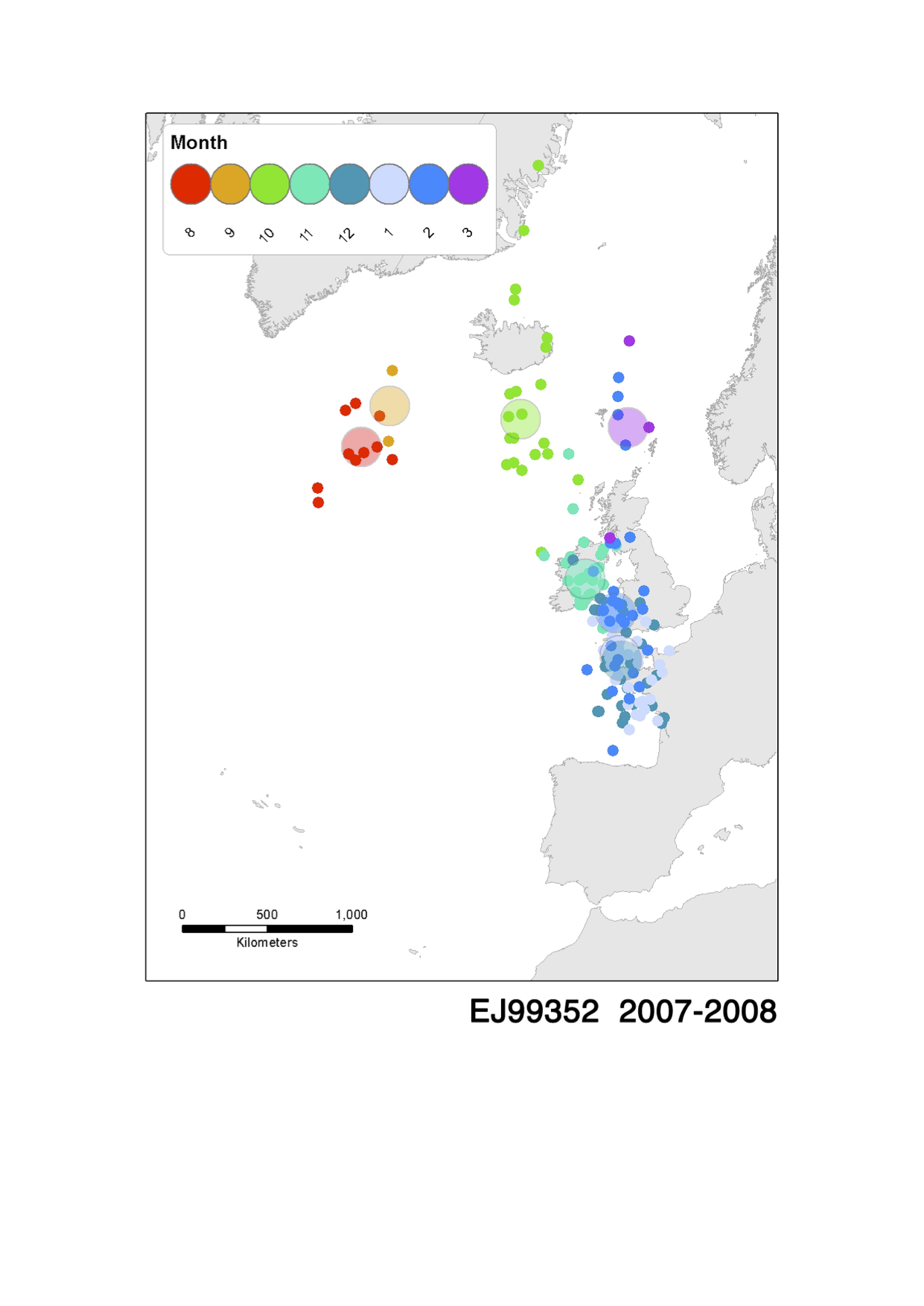

Supplement: Figure S2 — Filtered (valid) geolocator position estimates (small circles), and monthly spatial median positions (large circles) for Puffin EJ99352, colour coded by month during the 2007–2008 non-breeding season. (TIFF) [file pone.0021336.s002.tiff]

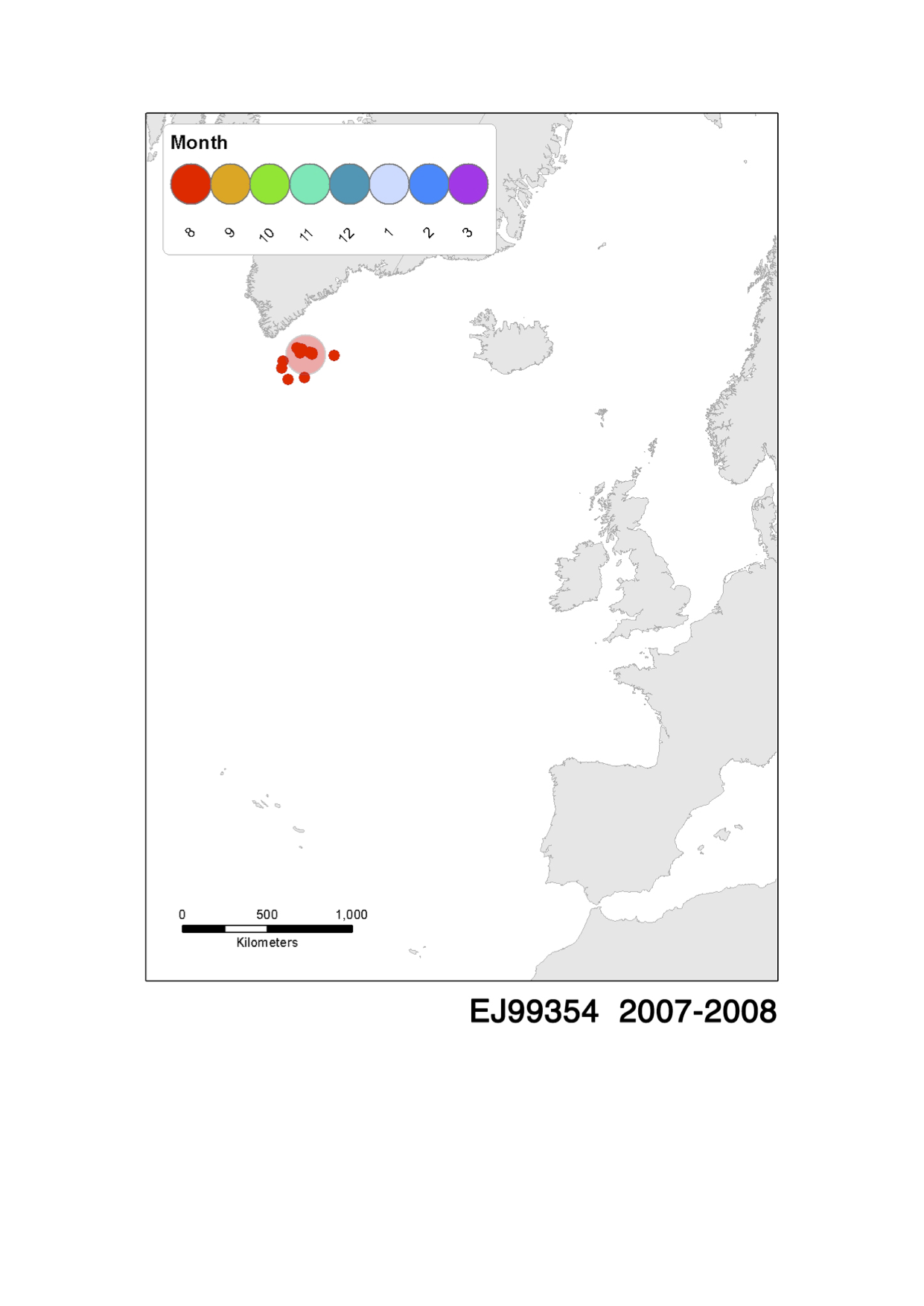

Supplement: Figure S3 — Filtered (valid) geolocator position estimates (small circles), and monthly spatial median positions (large circles) for Puffin EJ99354, colour coded by month during the 2007–2008 non-breeding season. (TIFF) [file pone.0021336.s003.tiff]

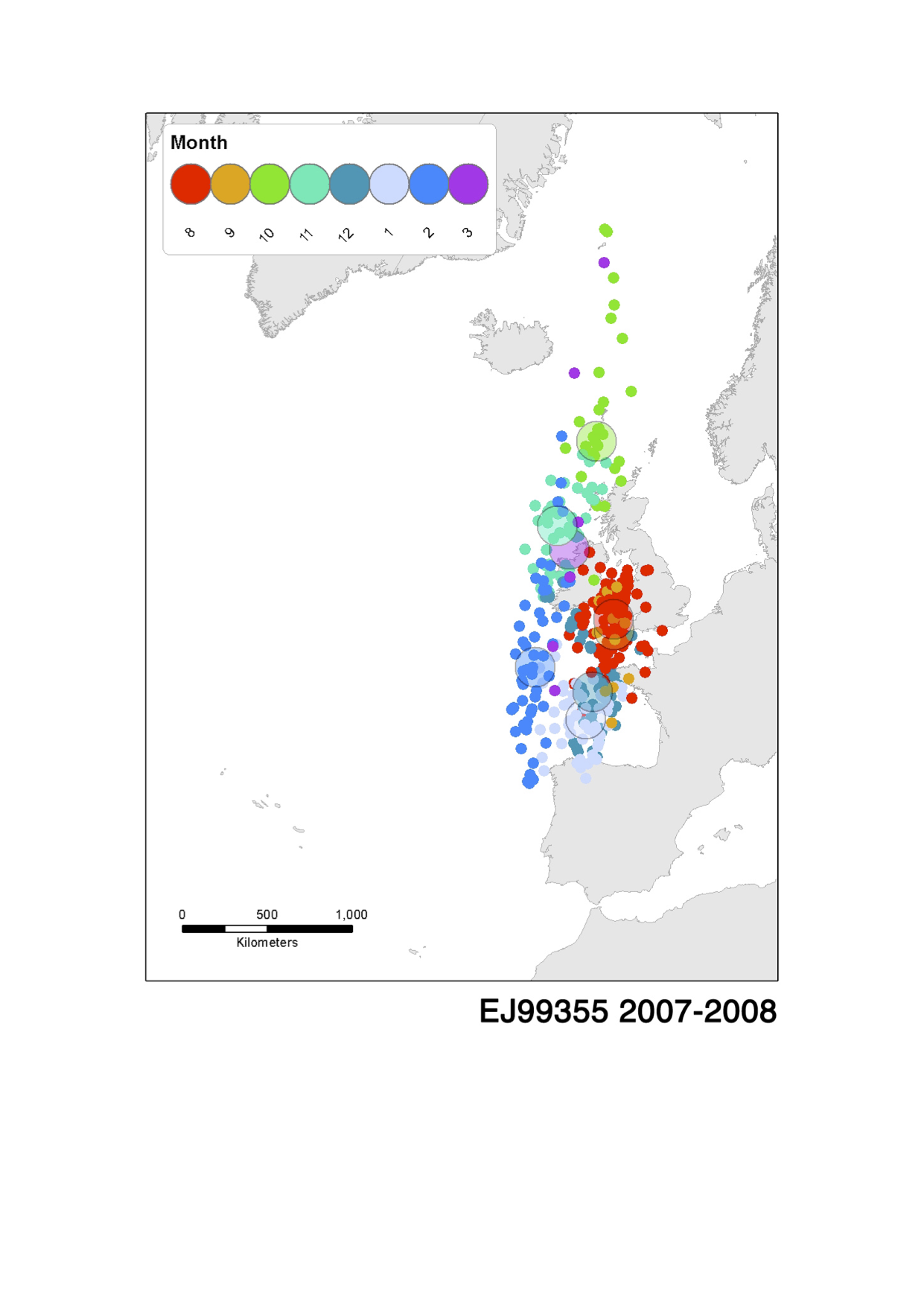

Supplement: Figure S4 — Filtered (valid) geolocator position estimates (small circles), and monthly spatial median positions (large circles) for Puffin EJ99355, colour coded by month during the 2007–2008 non-breeding season. (TIFF) [file pone.0021336.s004.tiff]

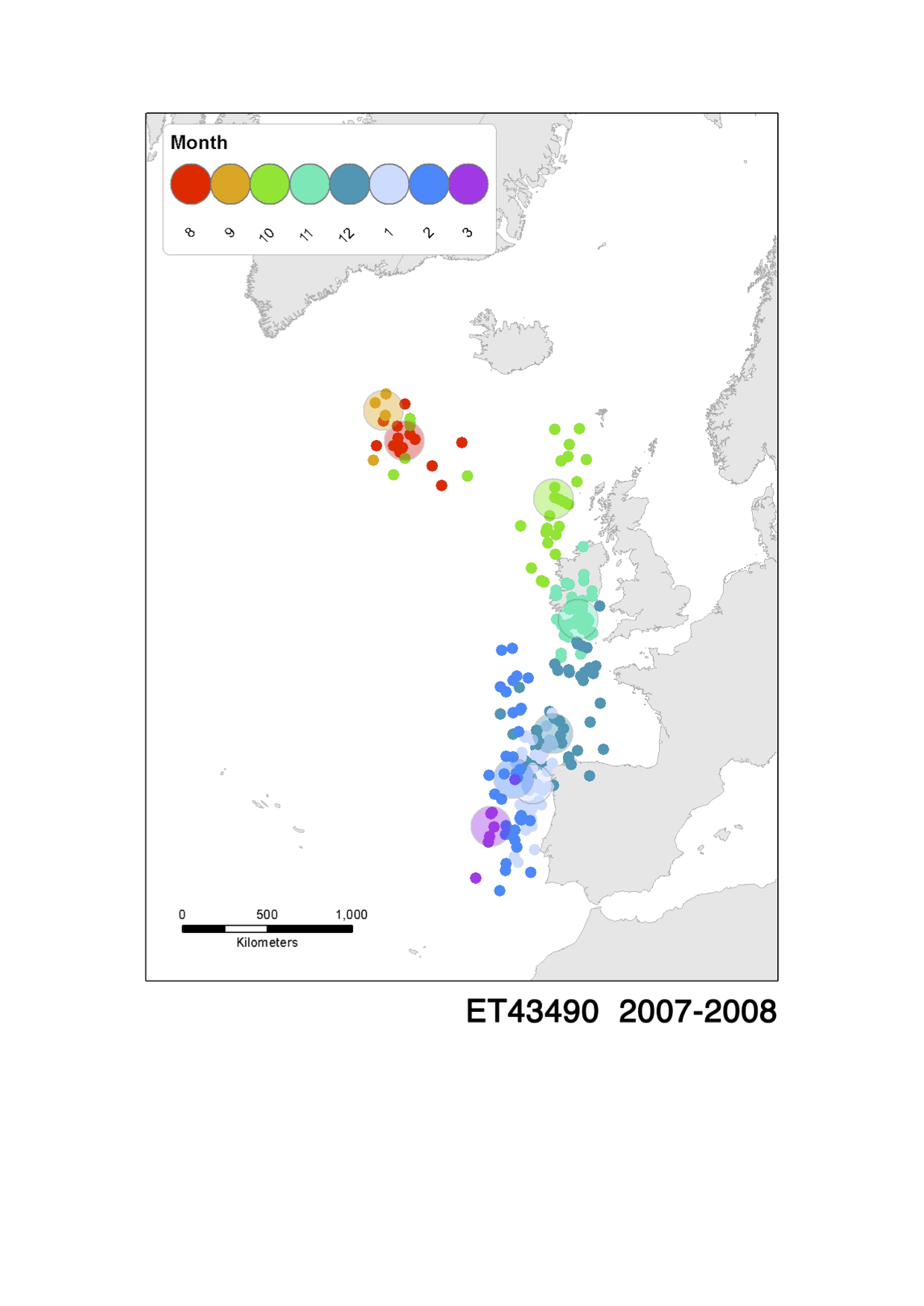

Supplement: Figure S5 — Filtered (valid) geolocator position estimates (small circles), and monthly spatial median positions (large circles) for Puffin ET43490, colour coded by month during the 2007–2008 non-breeding season. (TIFF) [file pone.0021336.s005.tiff]

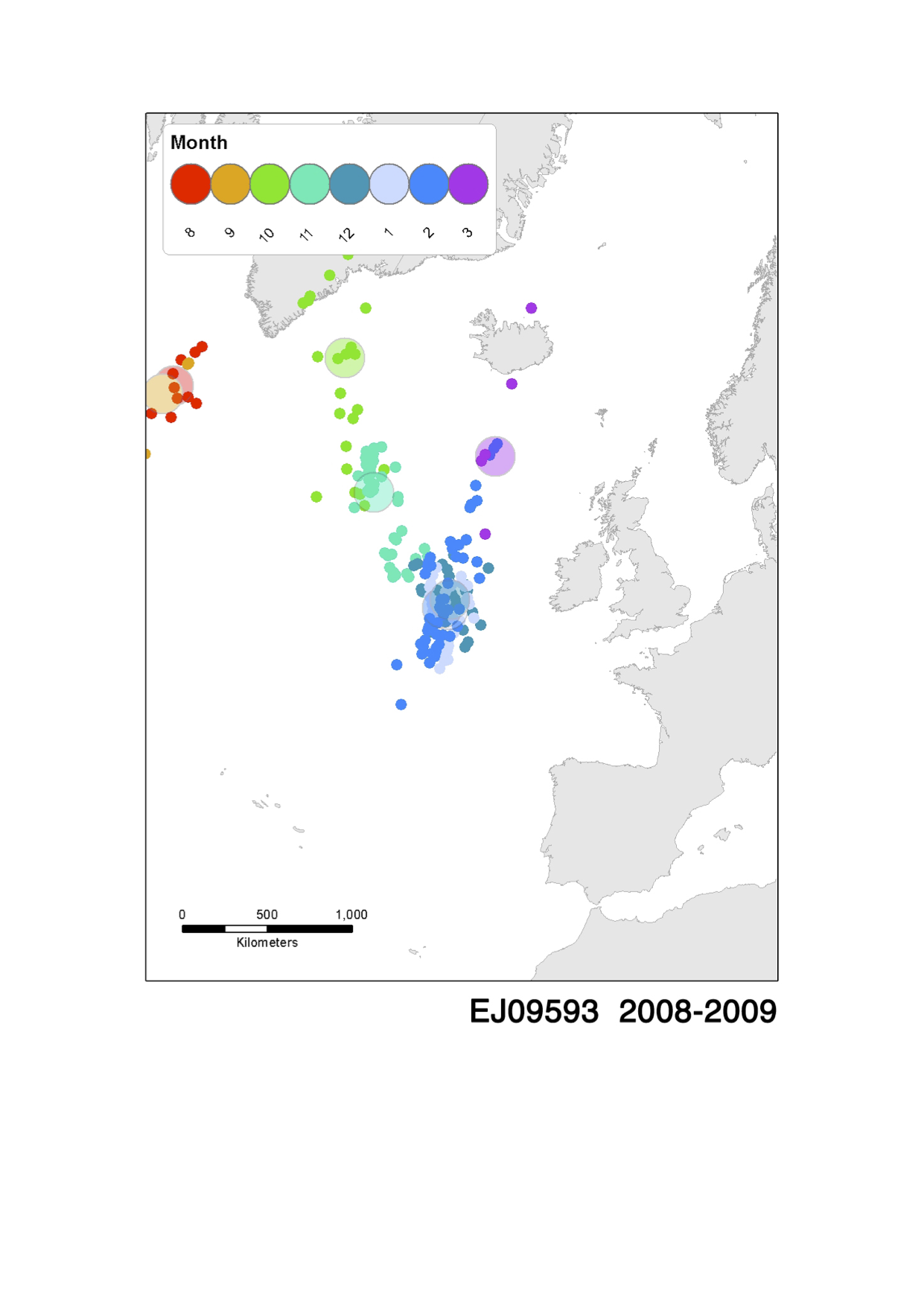

Supplement: Figure S6 — Filtered (valid) geolocator position estimates (small circles), and monthly spatial median positions (large circles) for Puffin EJ09593, colour coded by month during the 2008–2009 non-breeding season. (TIFF) [file pone.0021336.s006.tiff]

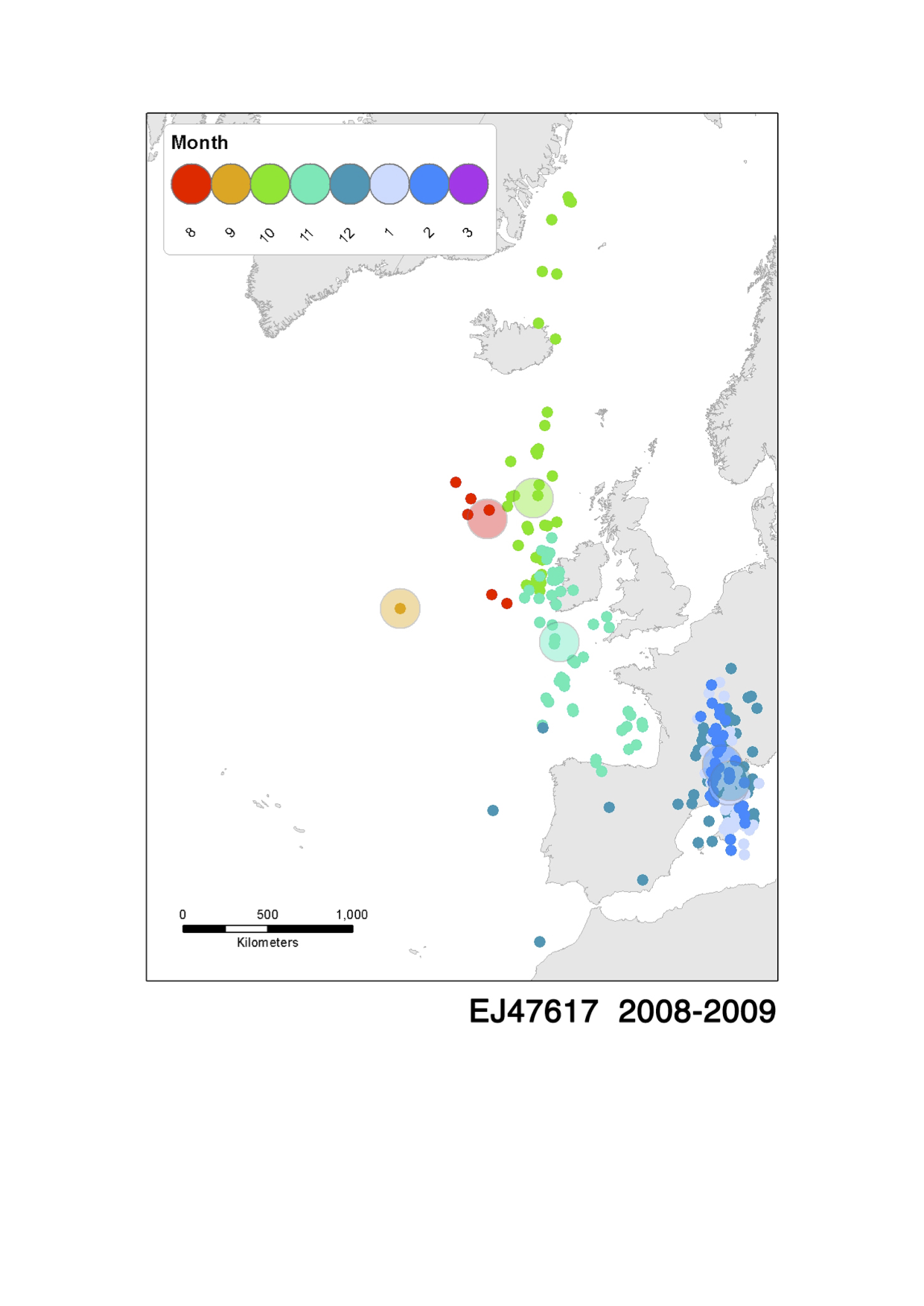

Supplement: Figure S7 — Filtered (valid) geolocator position estimates (small circles), and monthly spatial median positions (large circles) for Puffin EJ47617, colour coded by month during the 2008–2009 non-breeding season. (TIFF) [file pone.0021336.s007.tiff]

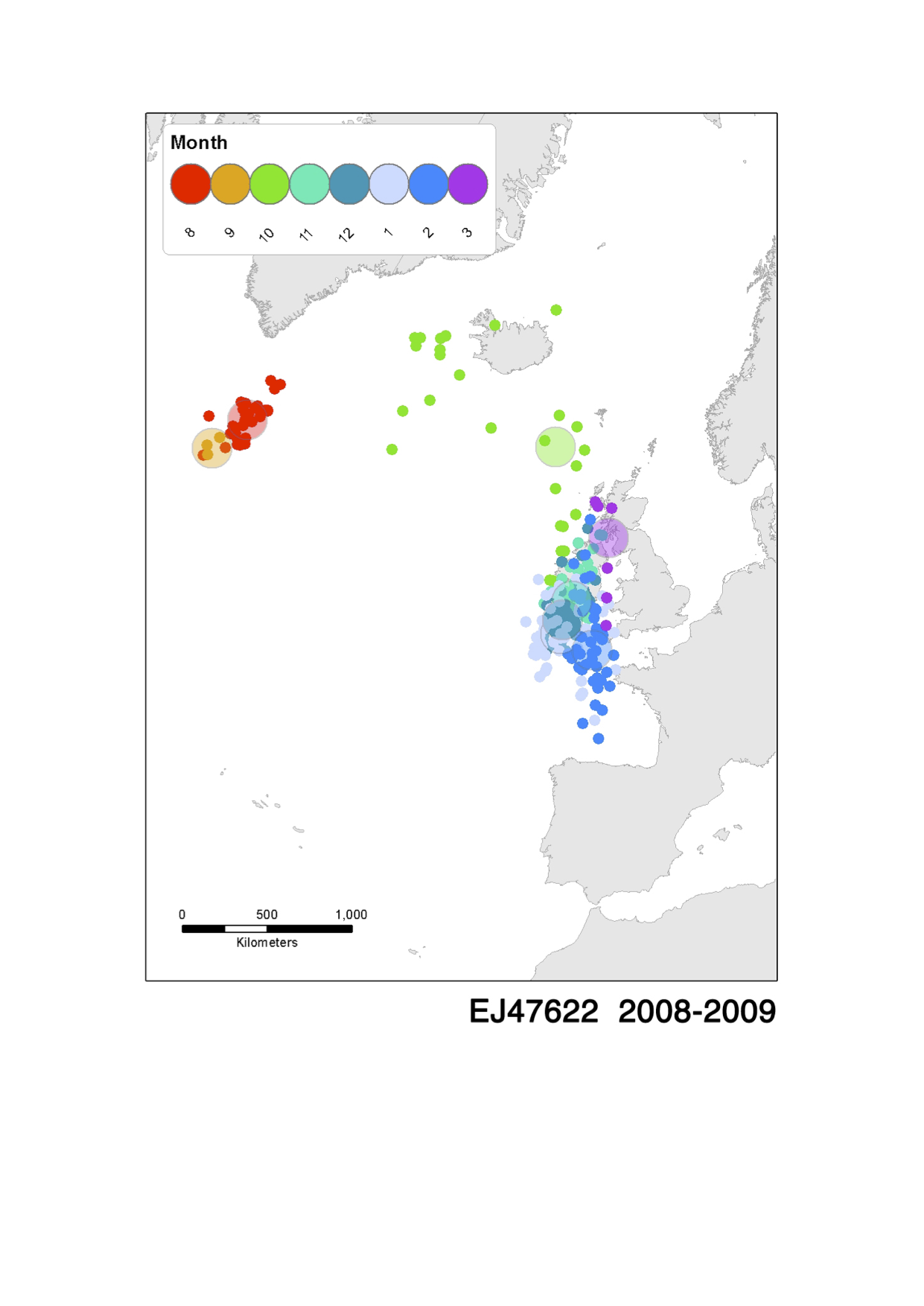

Supplement: Figure S8 — Filtered (valid) geolocator position estimates (small circles), and monthly spatial median positions (large circles) for Puffin EJ47622, colour coded by month during the 2008–2009 non-breeding season. (TIFF) [file pone.0021336.s008.tiff]

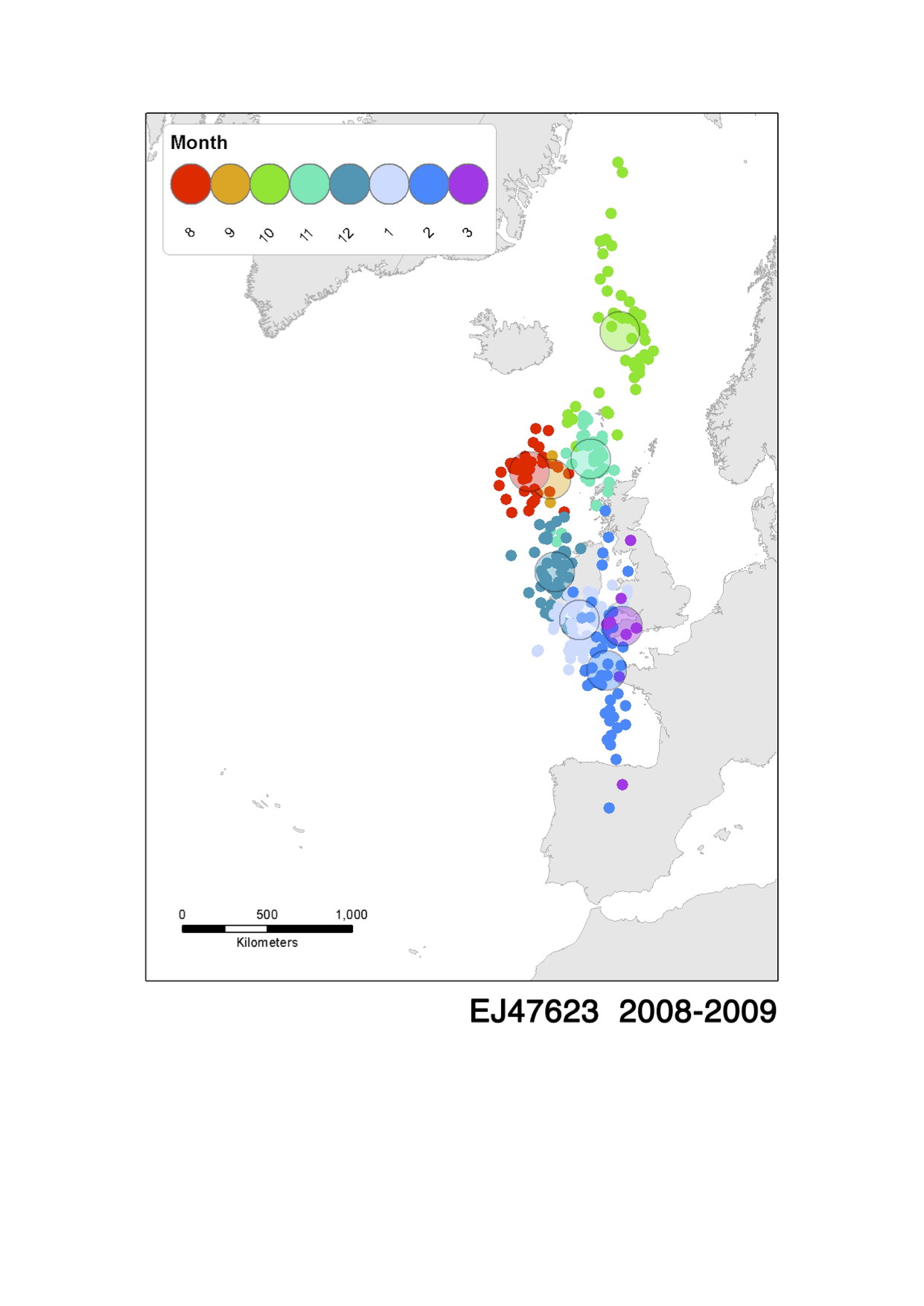

Supplement: Figure S9 — Filtered (valid) geolocator position estimates (small circles), and monthly spatial median positions (large circles) for Puffin EJ47623, colour coded by month during the 2008–2009 non-breeding season. (TIFF) [file pone.0021336.s009.tiff]

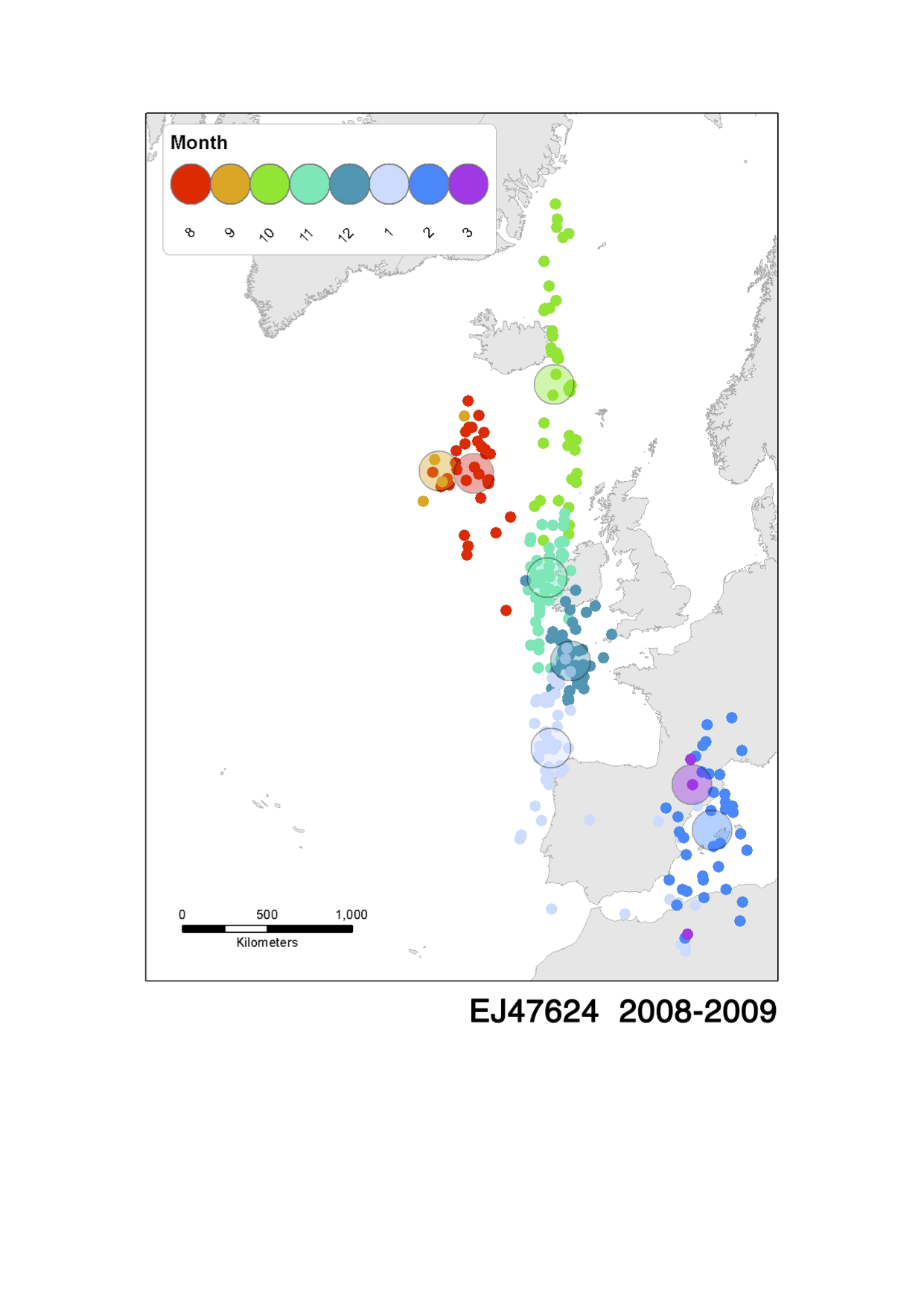

Supplement: Figure S10 — Filtered (valid) geolocator position estimates (small circles), and monthly spatial median positions (large circles) for Puffin EJ47624, colour coded by month during the 2008–2009 non-breeding season. (TIFF) [file pone.0021336.s010.tiff]

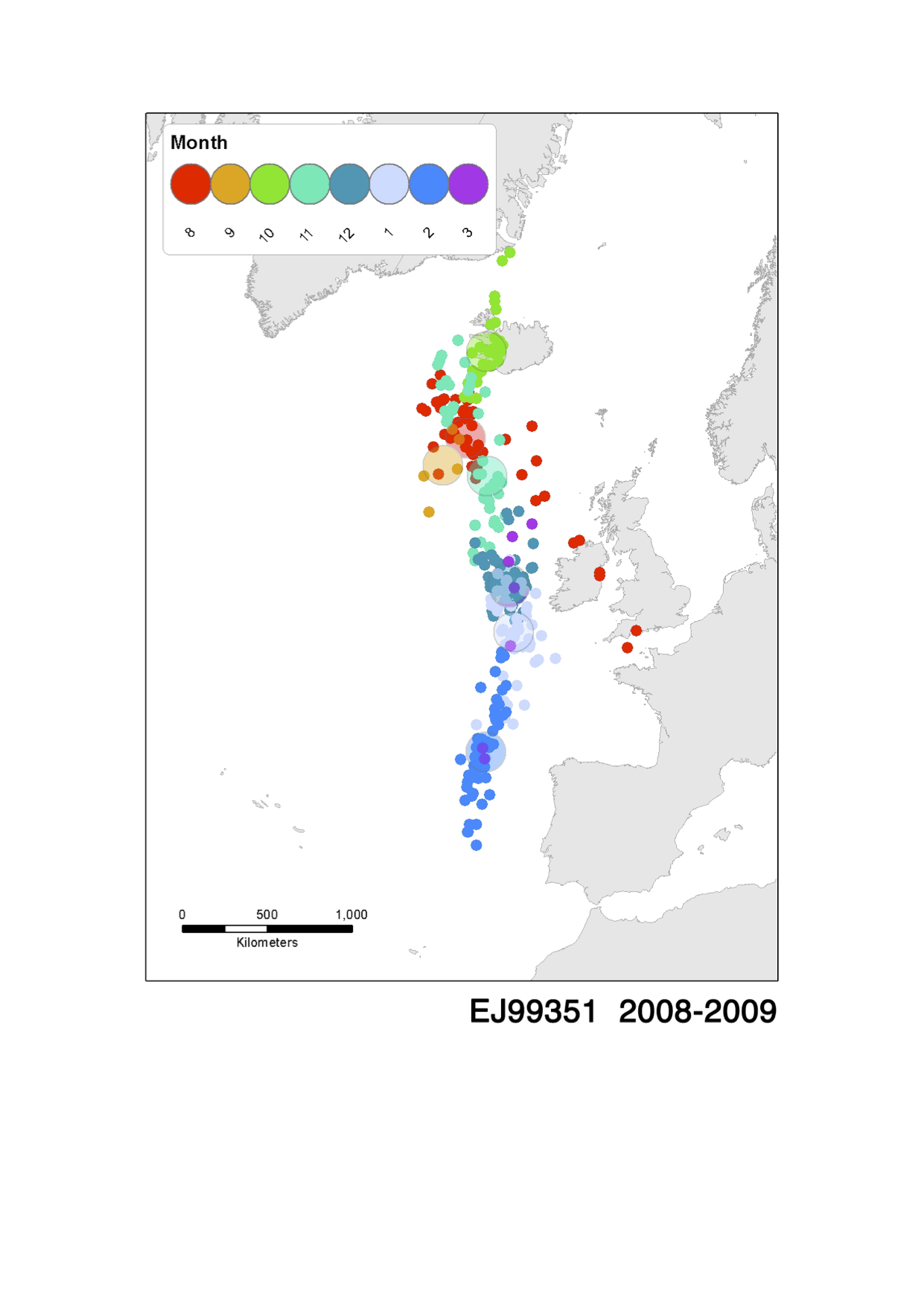

Supplement: Figure S11 — Filtered (valid) geolocator position estimates (small circles), and monthly spatial median positions (large circles) for Puffin EJ99351, colour coded by month during the 2008–2009 non-breeding season. (TIFF) [file pone.0021336.s011.tiff]

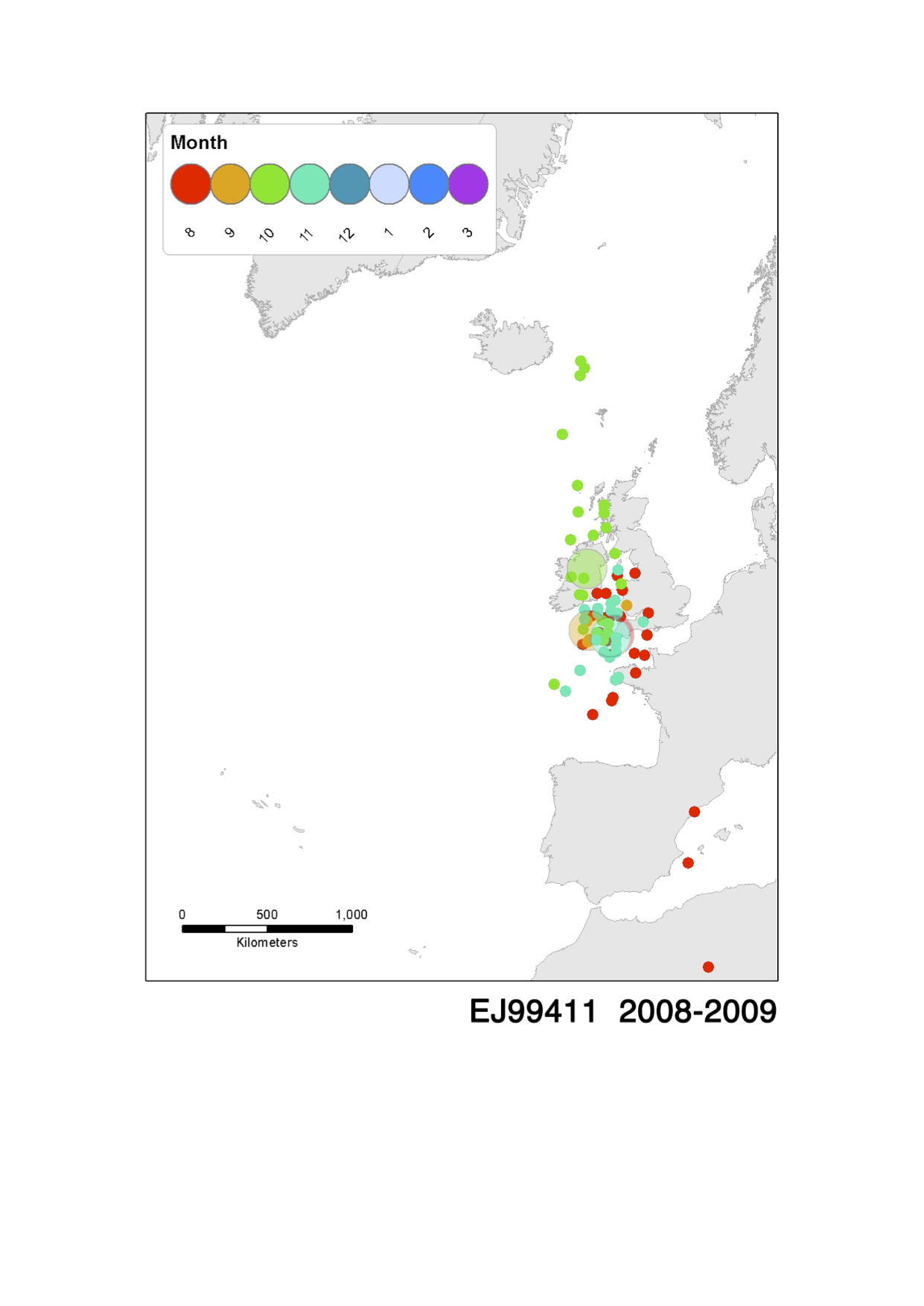

Supplement: Figure S12 — Filtered (valid) geolocator position estimates (small circles), and monthly spatial median positions (large circles) for Puffin EJ99411, colour coded by month during the 2008–2009 non-breeding season. (TIFF) [file pone.0021336.s012.tiff]

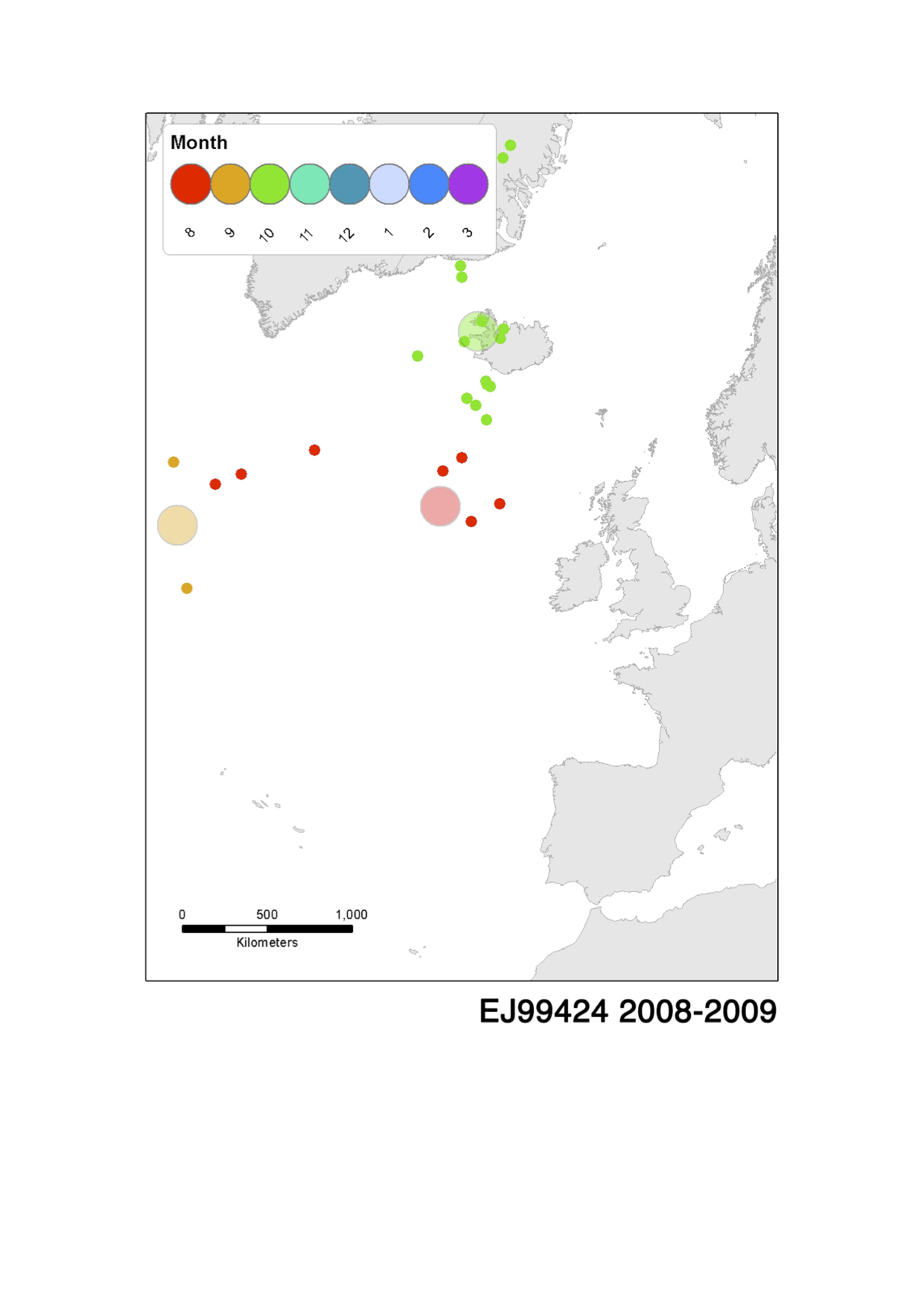

Supplement: Figure S13 — Filtered (valid) geolocator position estimates (small circles), and monthly spatial median positions (large circles) for Puffin EJ99424, colour coded by month during the 2008–2009 non-breeding season. (TIFF) [file pone.0021336.s013.tiff]

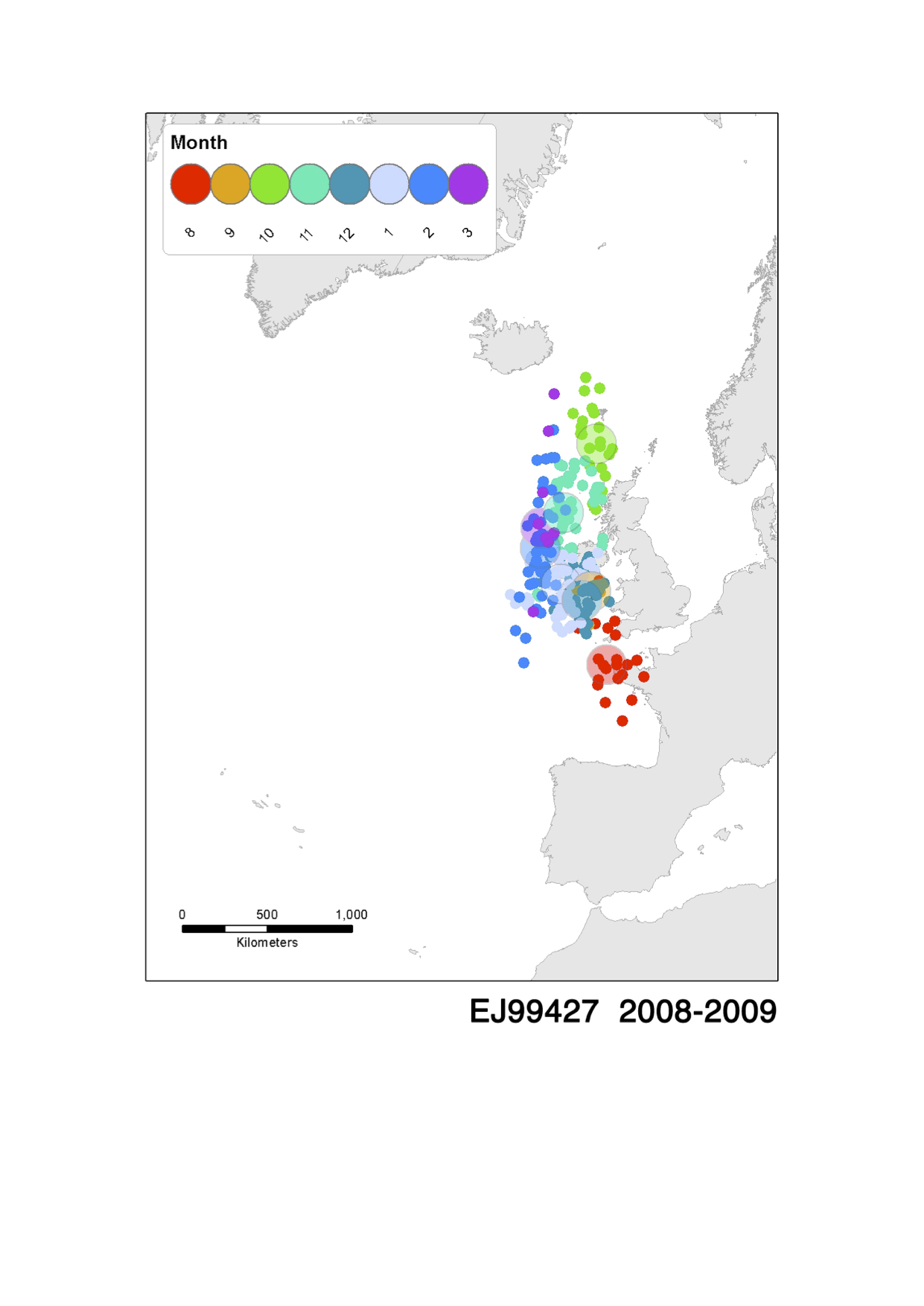

Supplement: Figure S14 — Filtered (valid) geolocator position estimates (small circles), and monthly spatial median positions (large circles) for Puffin EJ99427, colour coded by month during the 2008–2009 non-breeding season. (TIFF) [file pone.0021336.s014.tiff]

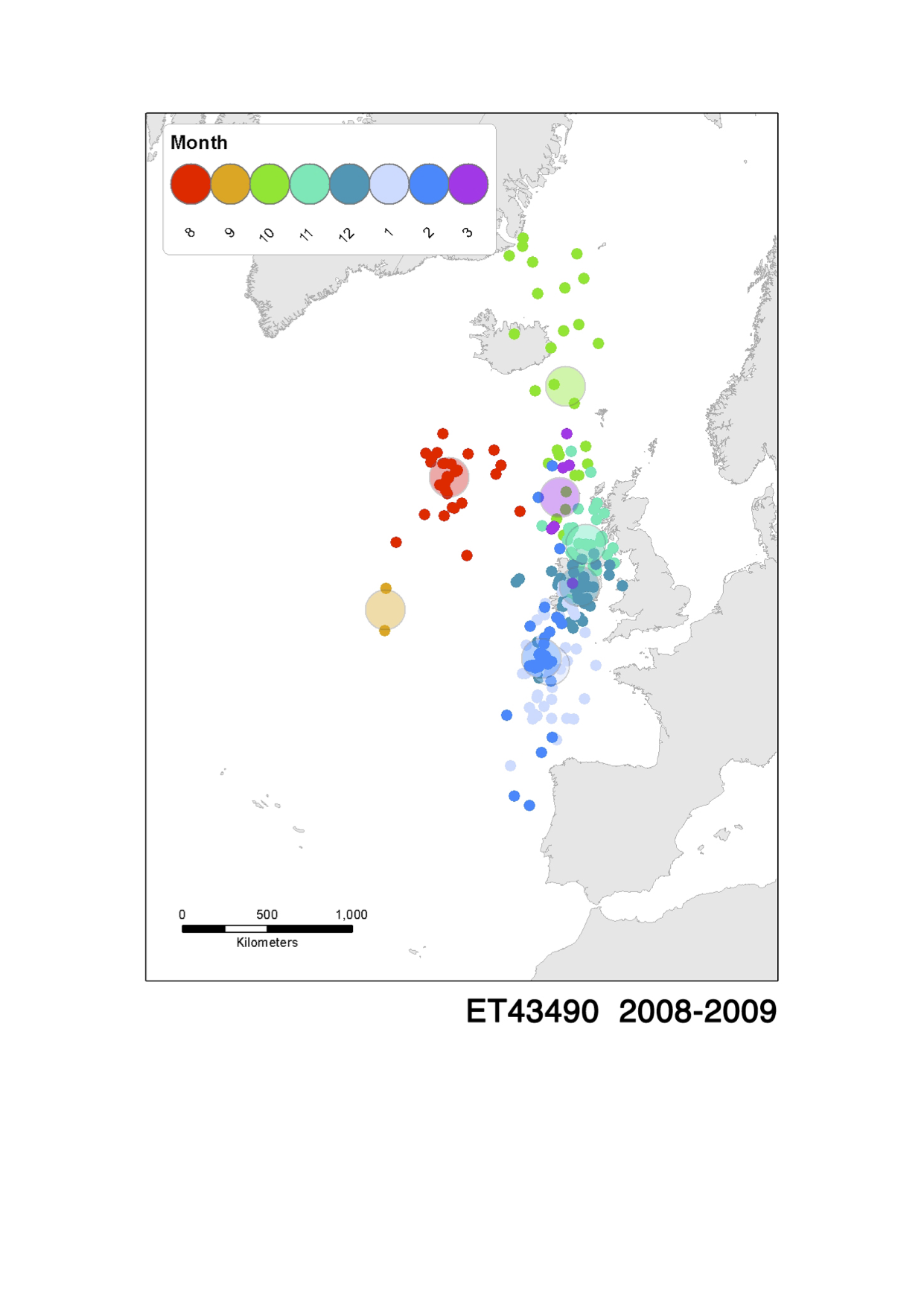

Supplement: Figure S15 — Filtered (valid) geolocator position estimates (small circles), and monthly spatial median positions (large circles) for Puffin EJ43490, colour coded by month during the 2008–2009 non-breeding season. (TIFF) [file pone.0021336.s015.tiff]

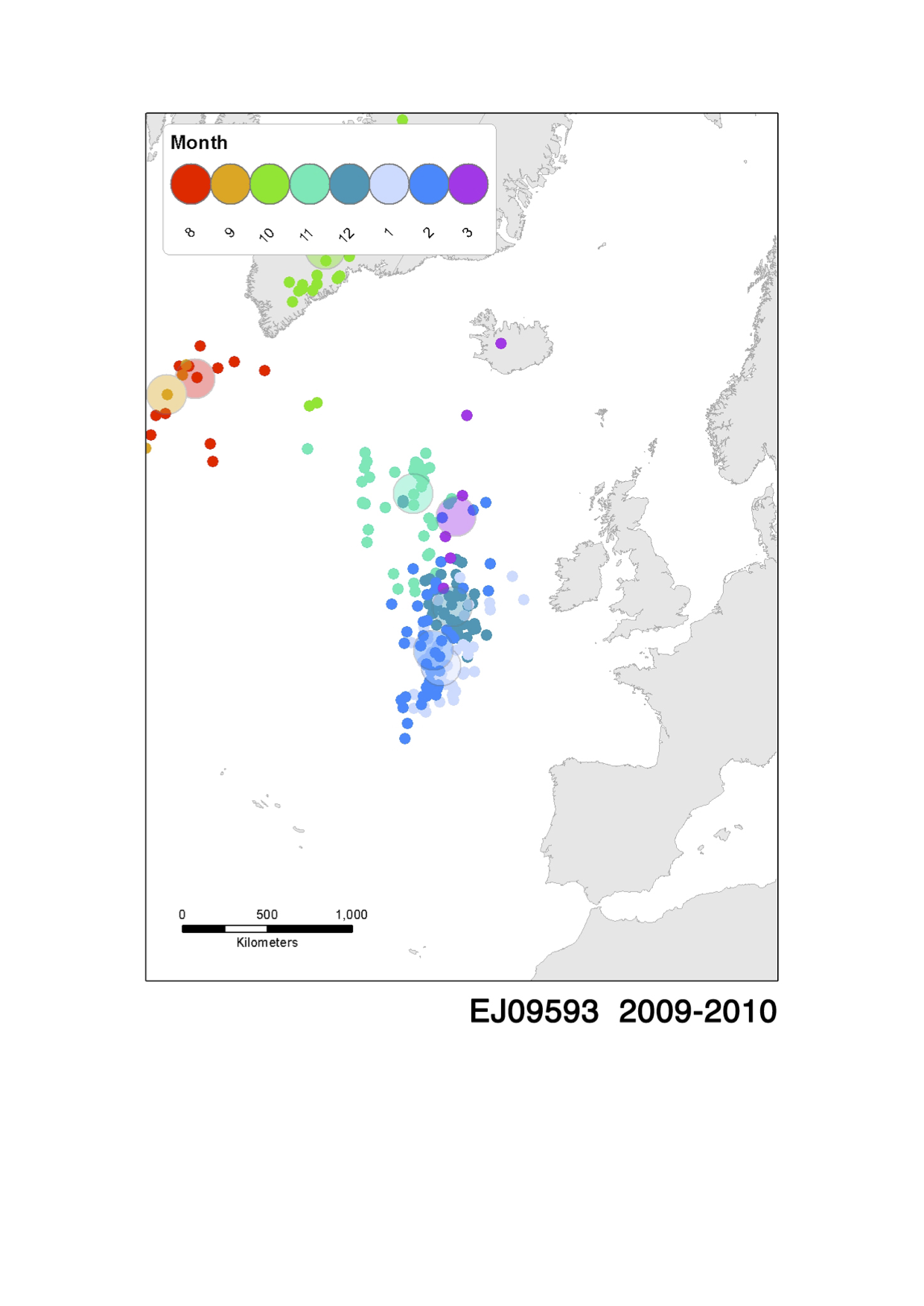

Supplement: Figure S16 — Filtered (valid) geolocator position estimates (small circles), and monthly spatial median positions (large circles) for Puffin EJ09593, colour coded by month during the 2009–2010 non-breeding season. (TIFF) [file pone.0021336.s016.tiff]

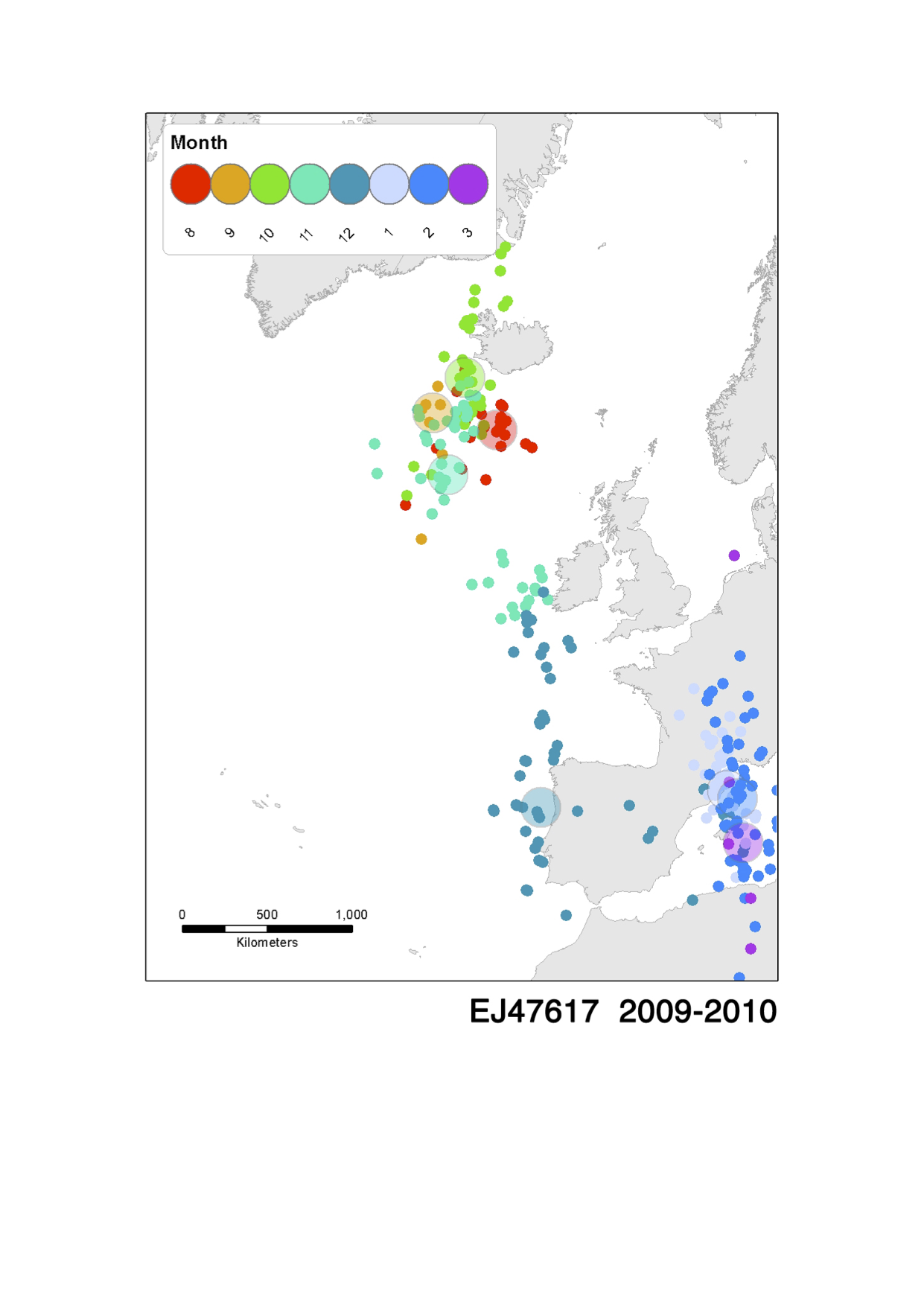

Supplement: Figure S17 — Filtered (valid) geolocator position estimates (small circles), and monthly spatial median positions (large circles) for Puffin EJ47617, colour coded by month during the 2009–2010 non-breeding season. (TIFF) [file pone.0021336.s017.tiff]

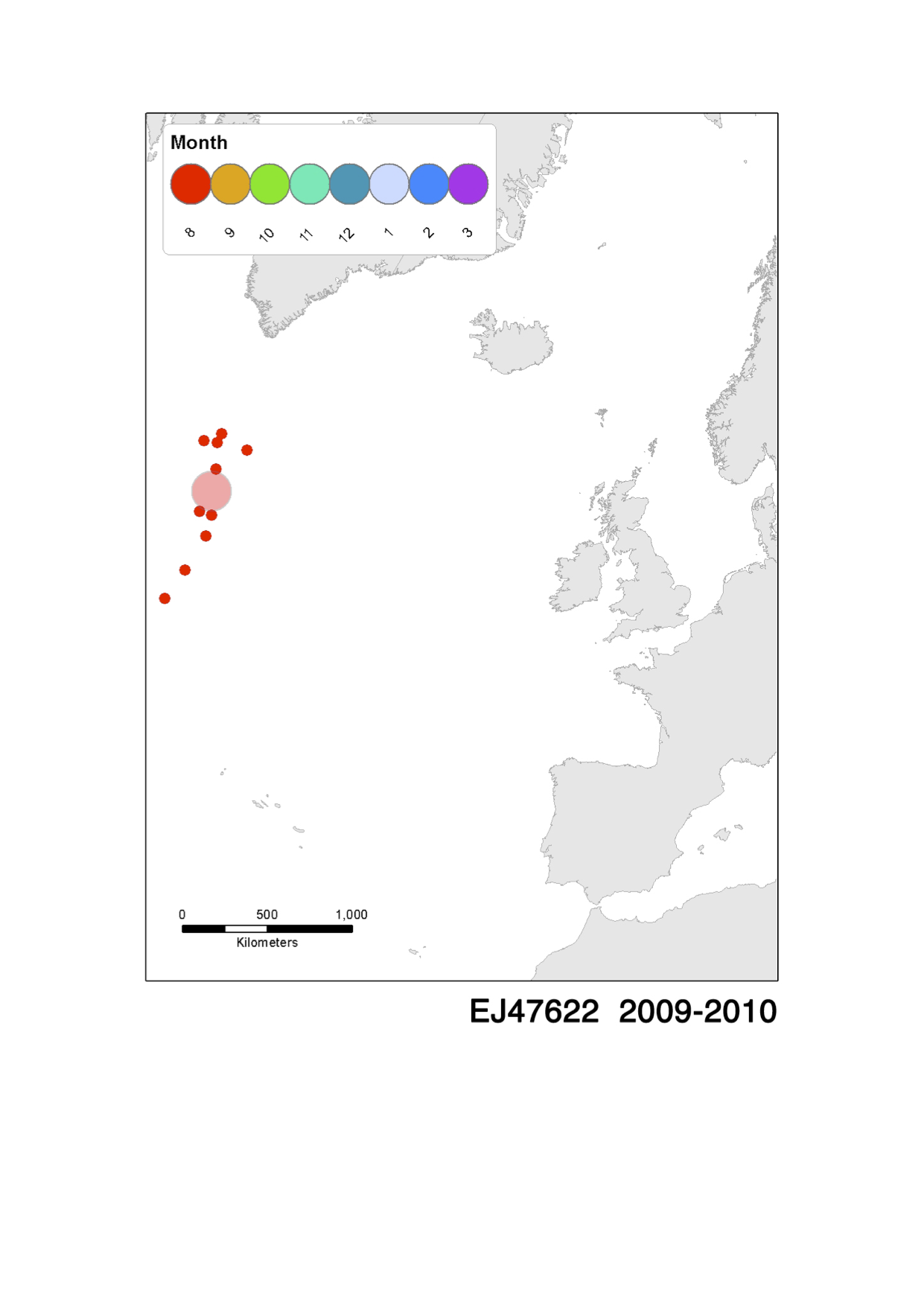

Supplement: Figure S18 — Filtered (valid) geolocator position estimates (small circles), and monthly spatial median positions (large circles) for Puffin EJ47622, colour coded by month during the 2009–2010 non-breeding season. (TIFF) [file pone.0021336.s018.tiff]

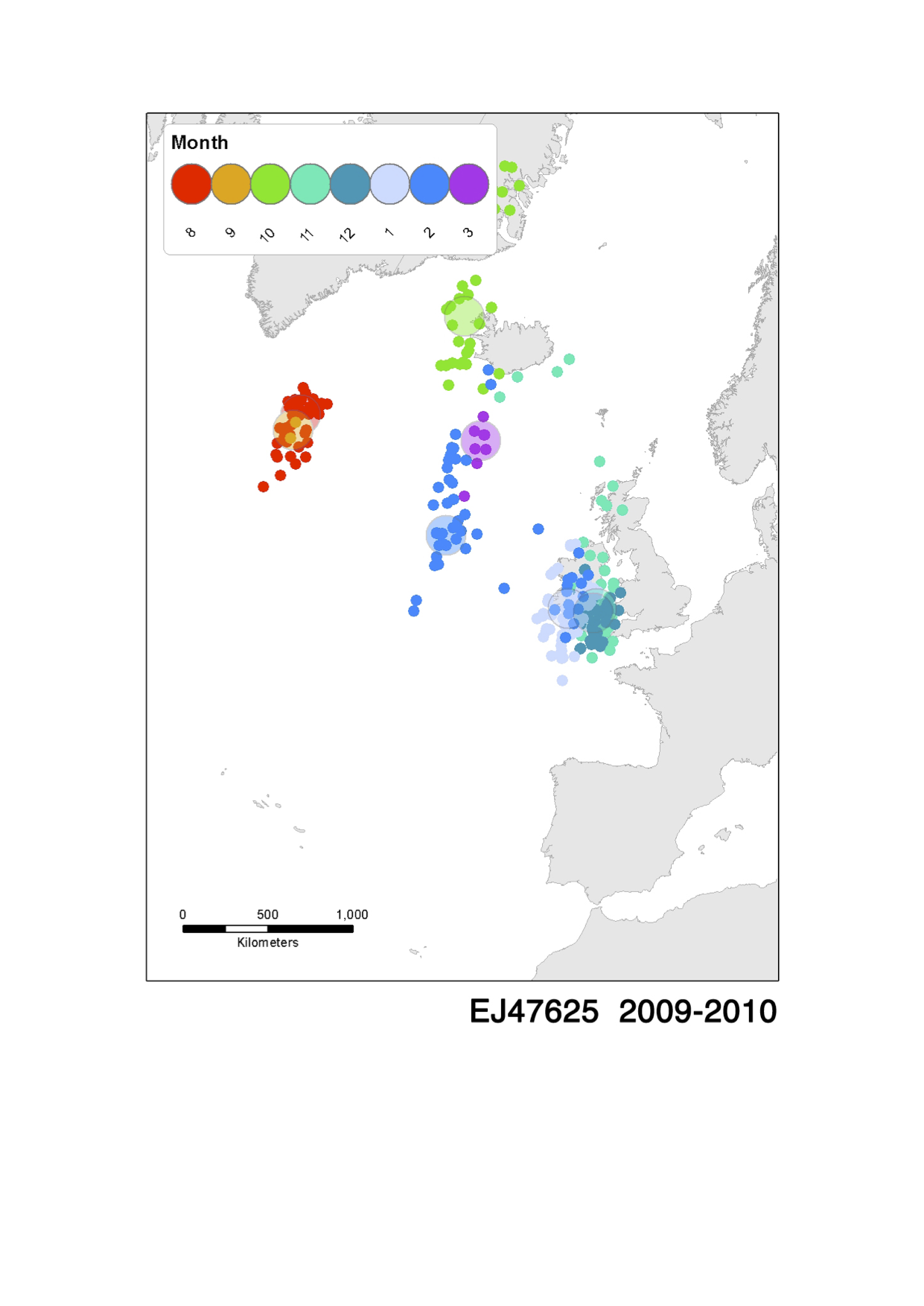

Supplement: Figure S19 — Filtered (valid) geolocator position estimates (small circles), and monthly spatial median positions (large circles) for Puffin EJ47625, colour coded by month during the 2009–2010 non-breeding season. (TIFF) [file pone.0021336.s019.tiff]

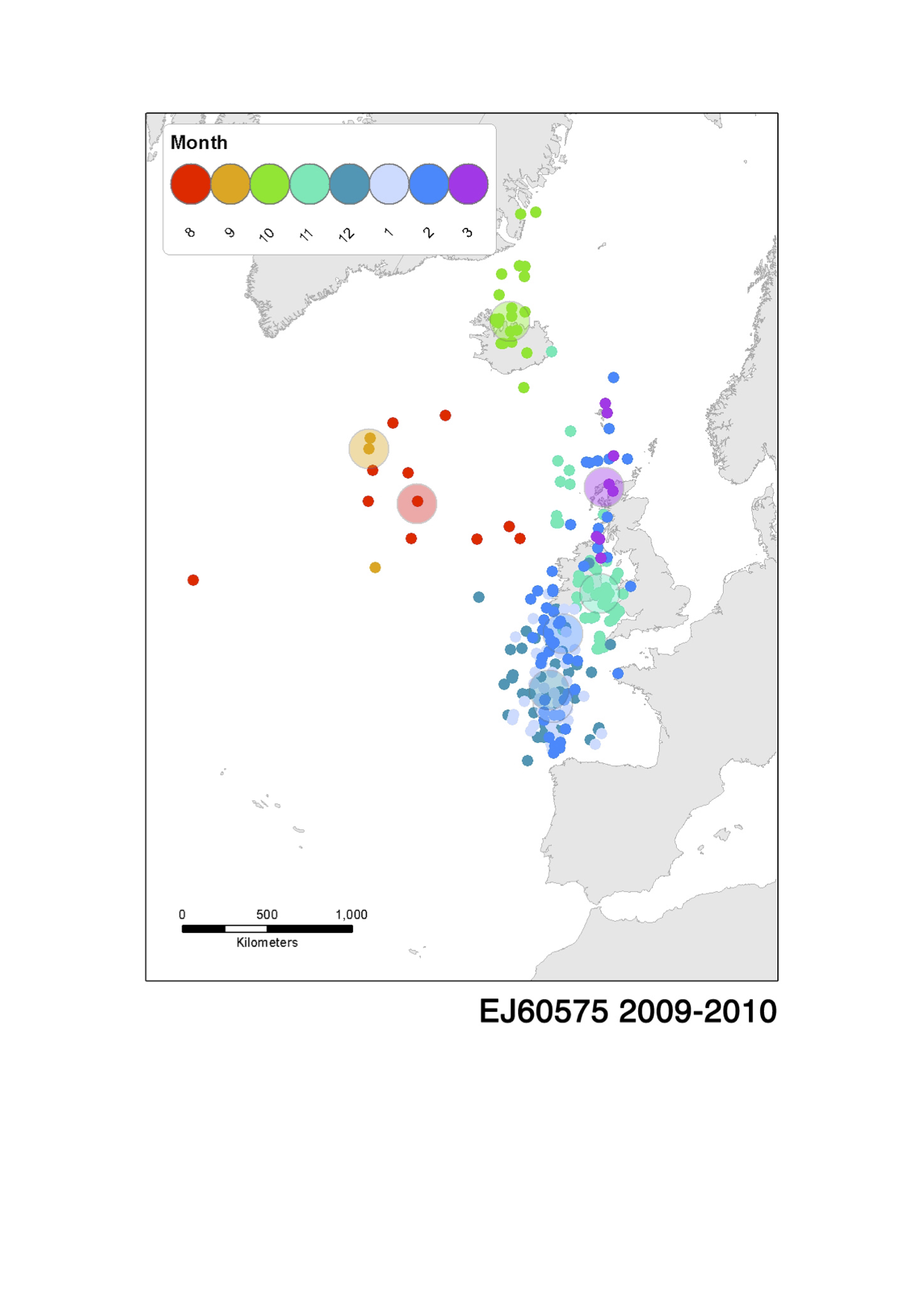

Supplement: Figure S20 — Filtered (valid) geolocator position estimates (small circles), and monthly spatial median positions (large circles) for Puffin EJ60575, colour coded by month during the 2009–2010 non-breeding season. (TIFF) [file pone.0021336.s020.tiff]

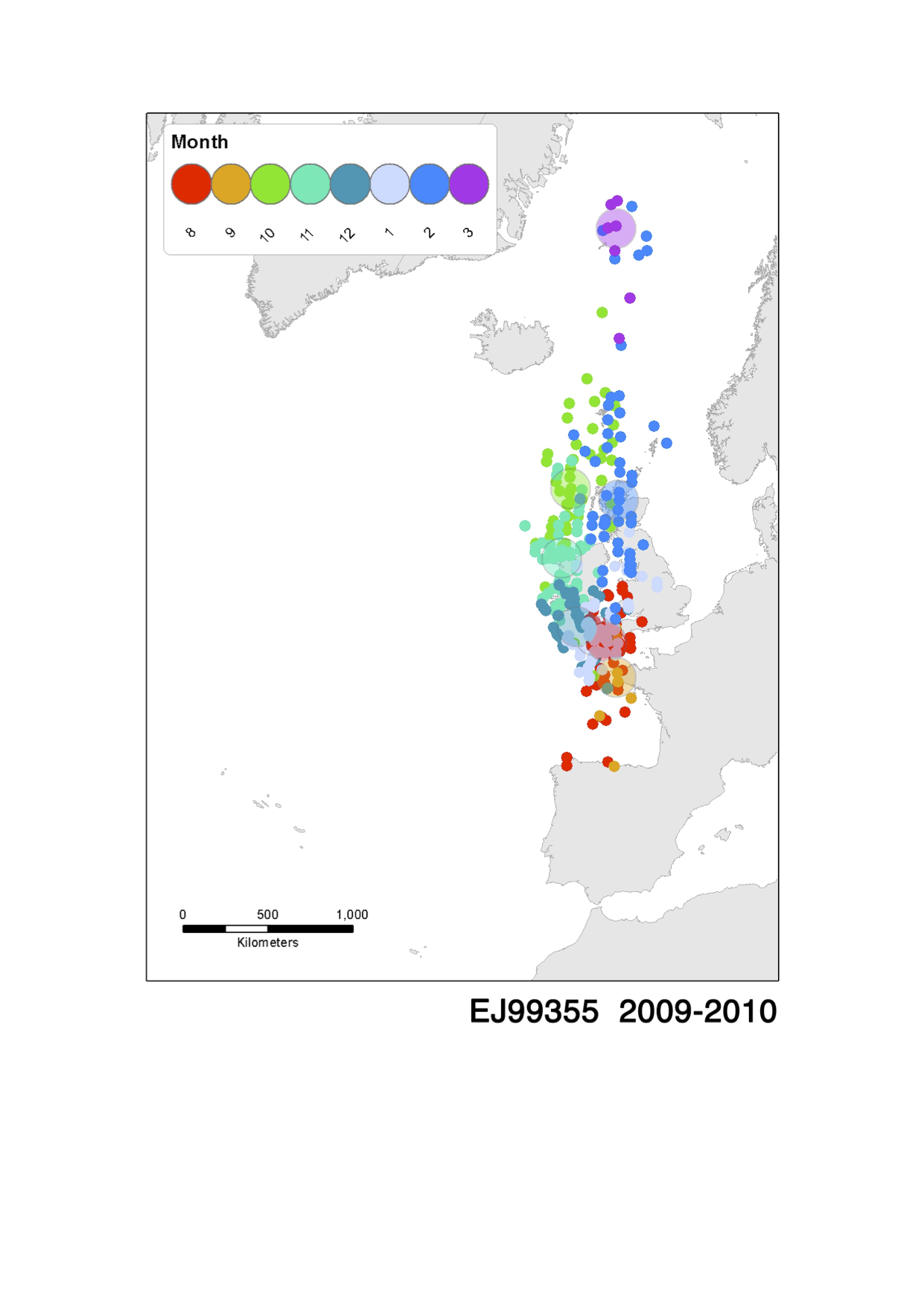

Supplement: Figure S21 — Filtered (valid) geolocator position estimates (small circles), and monthly spatial median positions (large circles) for Puffin EJ99355, colour coded by month during the 2009–2010 non-breeding season. (TIFF) [file pone.0021336.s021.tiff]

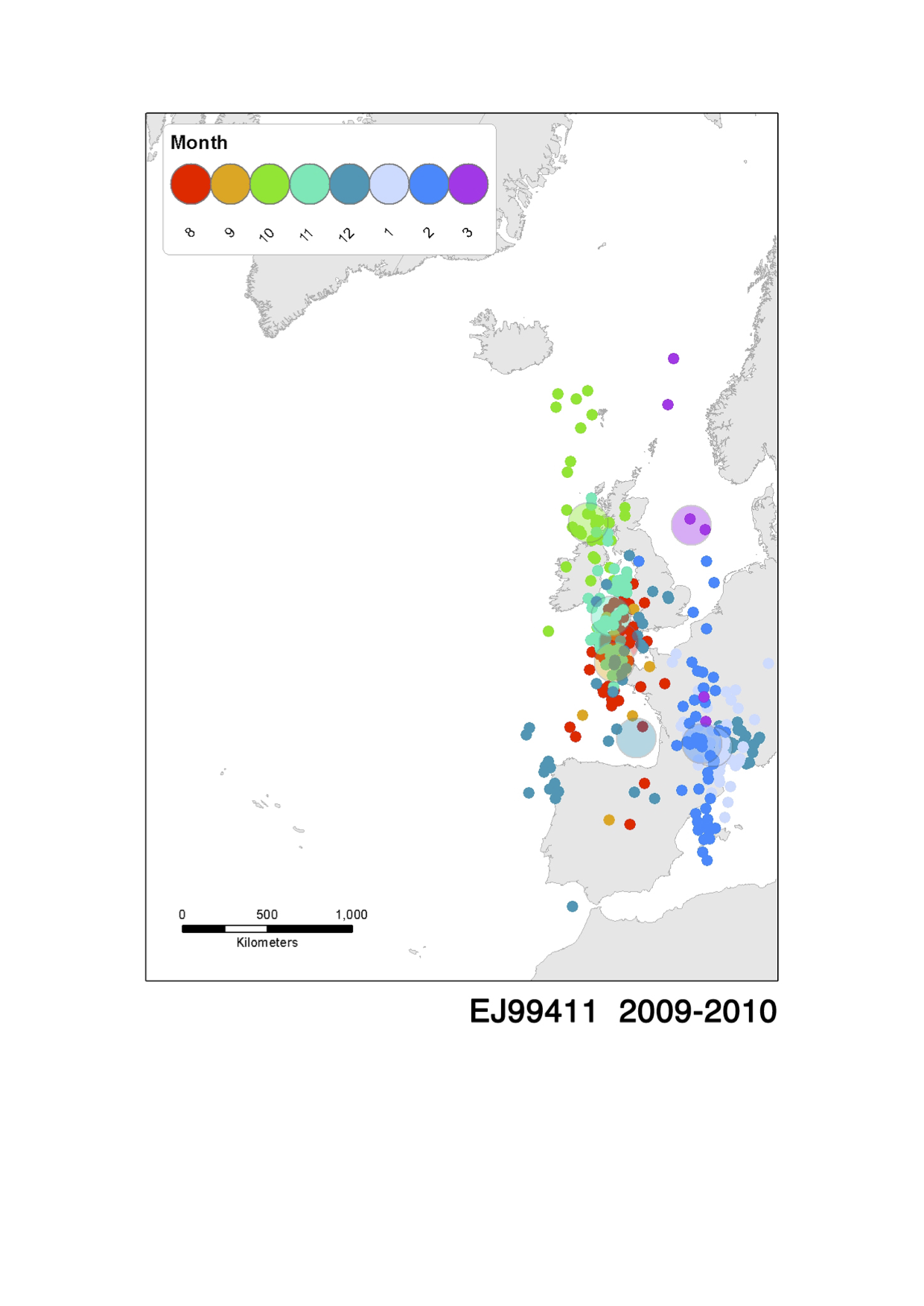

Supplement: Figure S22 — Filtered (valid) geolocator position estimates (small circles), and monthly spatial median positions (large circles) for Puffin EJ99411, colour coded by month during the 2009–2010 non-breeding season. (TIFF) [file pone.0021336.s022.tiff]

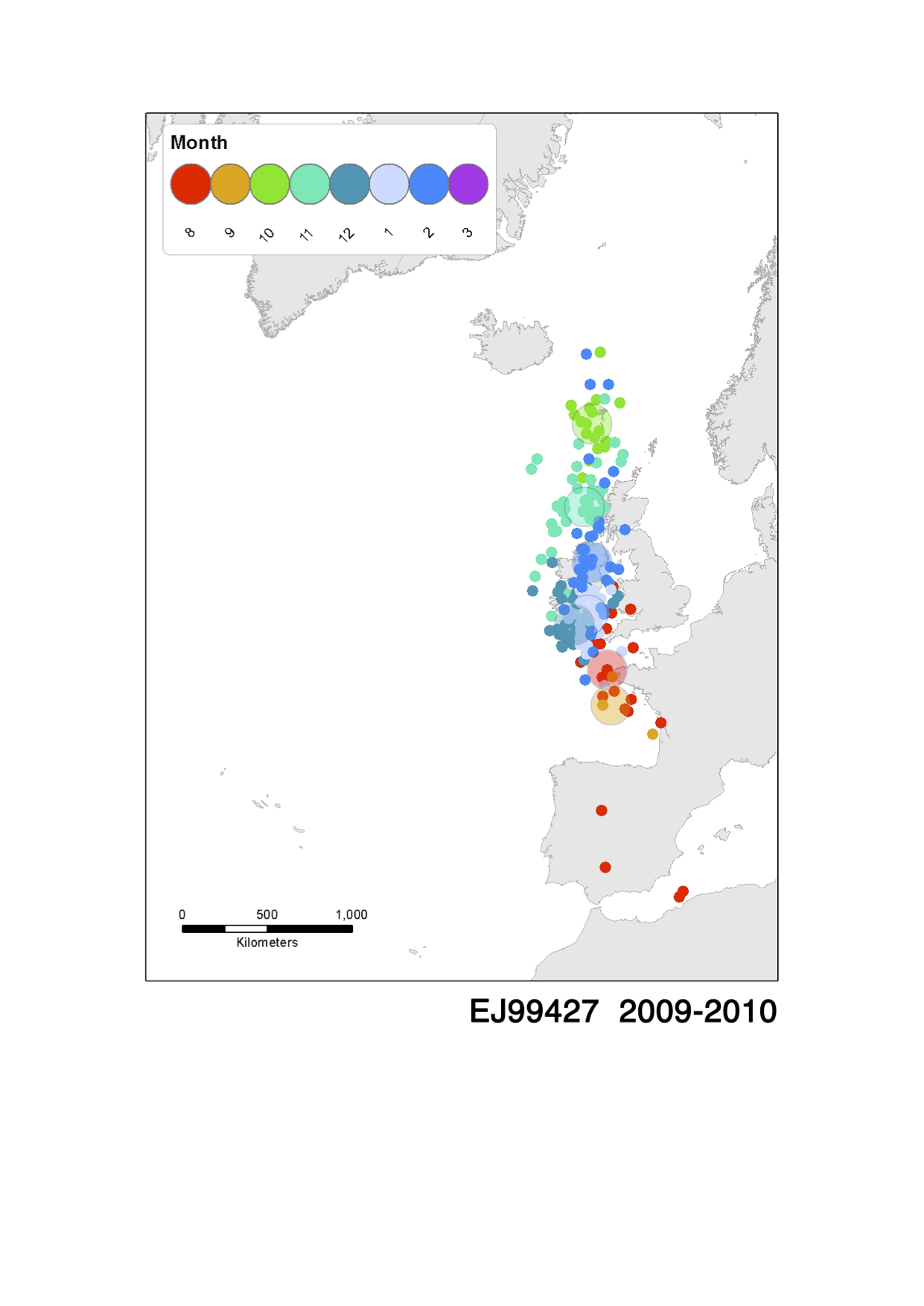

Supplement: Figure S23 — Filtered (valid) geolocator position estimates (small circles), and monthly spatial median positions (large circles) for Puffin EJ99427, colour coded by month during the 2009–2010 non-breeding season. (TIFF) [file pone.0021336.s023.tiff]

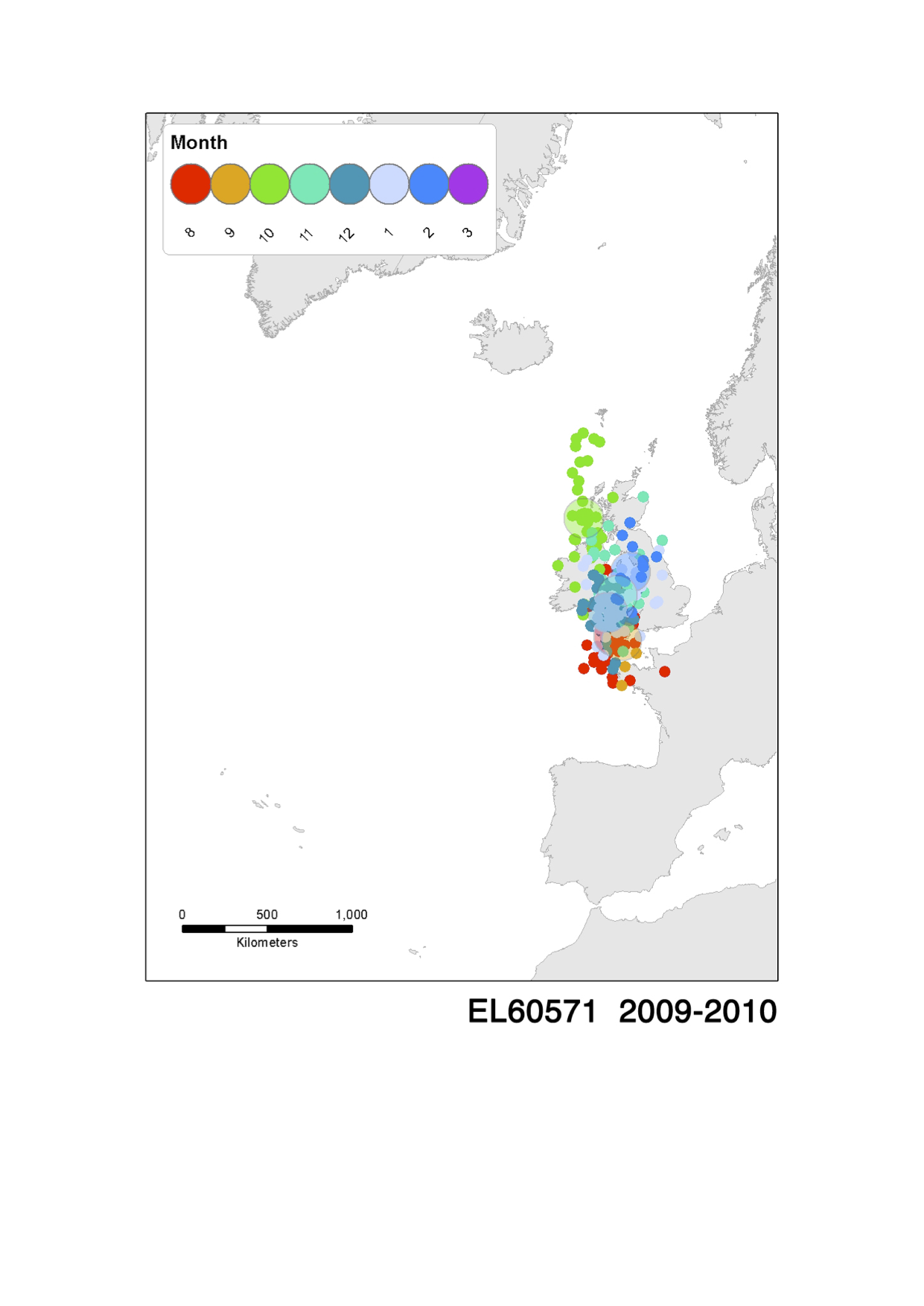

Supplement: Figure S24 — Filtered (valid) geolocator position estimates (small circles), and monthly spatial median positions (large circles) for Puffin EL60571, colour coded by month during the 2009–2010 non-breeding season. (TIFF) [file pone.0021336.s024.tiff]

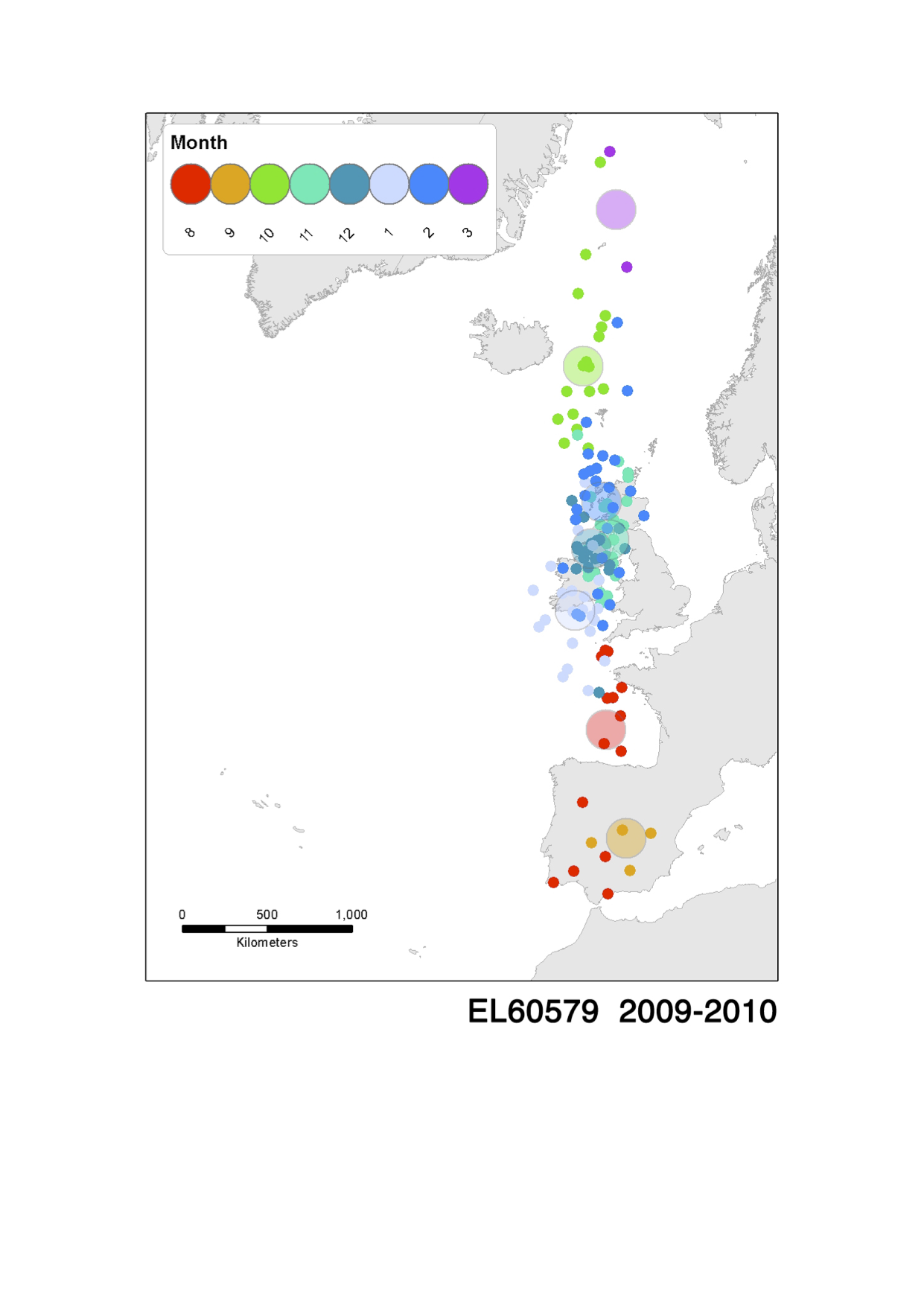

Supplement: Figure S25 — Filtered (valid) geolocator position estimates (small circles), and monthly spatial median positions (large circles) for Puffin EL60579, colour coded by month during the 2009–2010 non-breeding season. (TIFF) [file pone.0021336.s025.tiff]

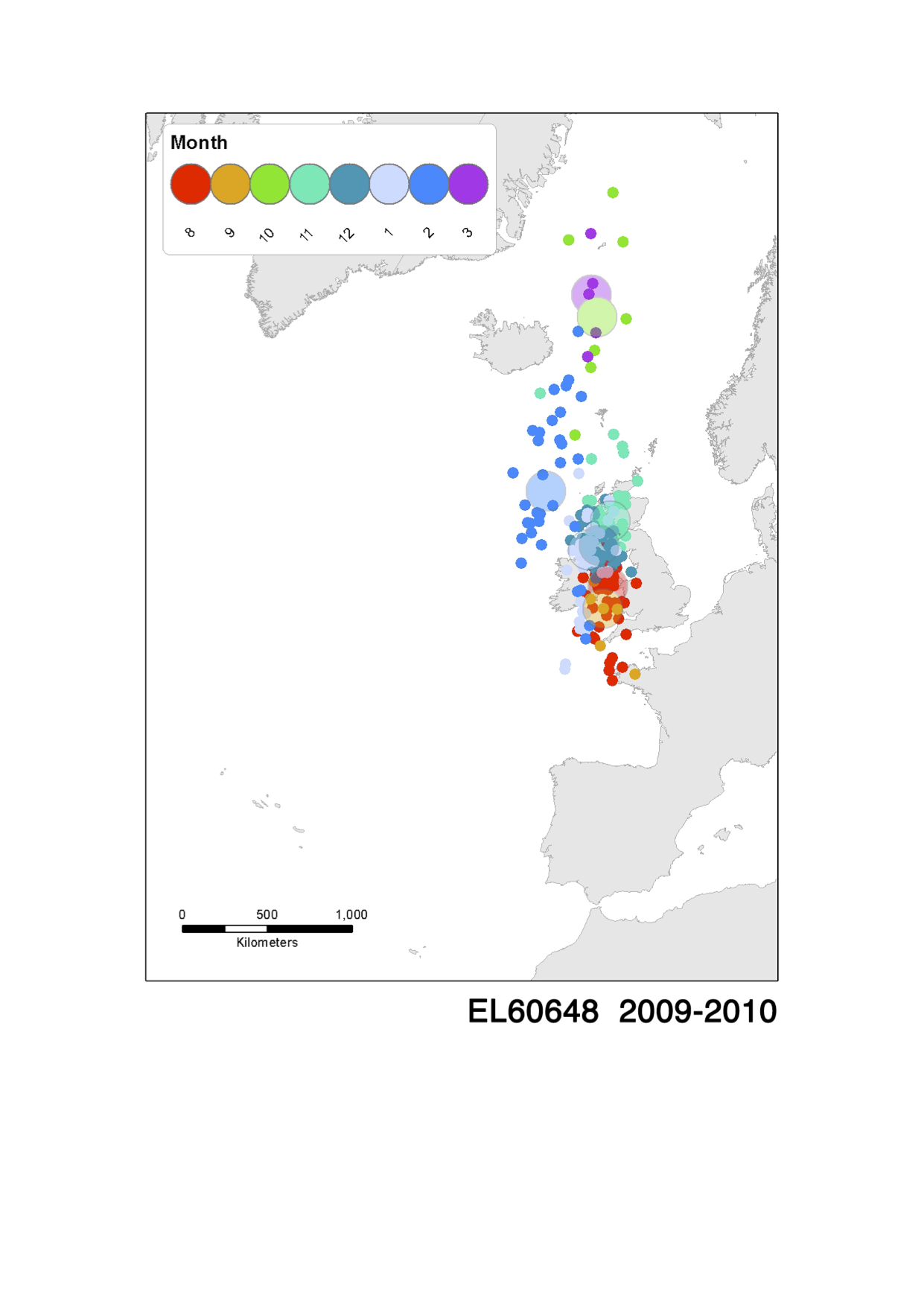

Supplement: Figure S26 — Filtered (valid) geolocator position estimates (small circles), and monthly spatial median positions (large circles) for Puffin EL60648, colour coded by month during the 2009–2010 non-breeding season. (TIFF) [file pone.0021336.s026.tiff]
