# Supplementary material for: A Dispersive Migration in the Atlantic Puffin and Its Implications for Migratory Navigation
Source: PLoS One. 2011 Jul 20;6(7):e21336. doi: 10.1371/journal.pone.0021336 (PMC3140476; doi:10.1371/journal.pone.0021336)
Supplement: Table S1 — Details of each individual Puffin used in the study including geolocator deployment periods, recaptures, the fate of geolocator data, the supplementary figure numbers containing displaying these data, and the breeding success of the bird in the season following deployment. (DOC) [file pone.0021336.s027.doc]

| **Bird ID** | **Deployment** | **Bird’s Fate** | **GLS data** | **Supp. Fig.** | **Breeding** |
| --- | --- | --- | --- | --- | --- |
| EL60569 | 2009/10 | Recaptured | Failed |  | Successful |
| EL60571 | 2009/10 | Recaptured | Full | SF24 | Successful |
| EL60572 | 2009/10 | Recaptured | Failed |  | Successful |
| EL60573 | 2009/10 | Recaptured | Failed |  | Successful |
| EL60575 | 2009/10 | Recaptured | Full | SF20 | Successful |
| EL60579 | 2009/10 | Recaptured | Full | SF25 | Successful |
| EL60648 | 2009/10 | Recaptured | Full | SF26 | Successful |
| EJ09593 | 2008/9 | Recaptured | Full | SF6 | Successful |
|  | 2009/10 | Recaptured | Full | SF16 | Successful |
| EJ99427 | 2008/9 | Recaptured | Full | SF14 | Successful |
|  | 2009/10 | Recaptured | Full | SF23 | Successful |
| EJ47617 | 2008/9 | Recaptured | Full | SF7 | Successful |
|  | 2009/10 | Recaptured | Full | SF17 | Successful |
| EJ47621 | 2008/9 | Seen |  |  | Failed |
|  | 2009/10 | Not Seen |  |  | Unknown |
| EJ47622 | 2008/9 | Seen | Full | SF8 | Successful |
|  | 2009/10 | Recaptured | Partial | SF18 | Successful |
| EJ47623 | 2007/8 | Recaptured | Failed |  | Successful |
|  | 2008/9 | Recaptured | Full | SF9 | Successful |
|  | 2009/10 | Seen |  |  | Successful |
| EJ47624 | 2008/9 | Recaptured | Full | SF10 | Successful |
|  | 2009/10 | Seen |  |  | Unknown |
| EJ47625 | 2008/9 | Recaptured | Failed |  | Successful |
|  | 2009/10 | Recaptured | Full | SF19 | Egg failed |
| EJ47626 | 2008/9 | Recaptured | Failed |  | Successful |
|  | 2009/10 | Recaptured | Failed |  | Successful |
| EJ99351 | 2007/8 | Seen | Full | SF1 | Failed |
|  | 2008/9 | Recaptured | Full | SF11 | Successful |
|  | 2009/10 | Recaptured | Failed |  | Successful |
| EJ99352 | 2007/8 | Recaptured | Full | SF2 | Successful |
|  | 2008/9 | Seen |  |  | Failed |
|  | 2009/10 | Seen |  |  | Chick failed |
| EJ99354 | 2007/8 | Recaptured | Partial | SF3 | Successful |
| EJ99355 | 2007/8 | Seen | Full | SF4 | Unknown |
|  | 2008/9 | Recaptured | Partial | Not used | Successful |
|  | 2009/10 | Recaptured | Full | SF21 | Successful |
| EJ99411 | 2008/9 | Recaptured | Partial | SF12 | Successful |
|  | 2009/10 | Recaptured | Full | SF22 | Successful |
| EJ99416 | 2008/9 | Seen |  |  | Failed |
|  | 2009/10 | Not seen |  |  | Unknown |
| EJ99417 | 2008/9 | Recaptured | Failed |  | Successful |
|  | 2009/10 | Recaptured | Failed |  | Successful |
| EJ99419 | 2009/10 | Not Seen |  |  | Unknown |
| EJ99420 | 2008/9 | Not Seen |  |  | Successful |
|  | 2009/10 | Seen |  |  | Successful |
| EJ99424 | 2008/9 | Seen | Partial | SF13 | Failed |
|  | 2009/10 | Recaptured | Failed |  | Successful |
| ET43490 | 2007/8 | Recaptured | Full | SF5 | Successful |
|  | 2008/9 | Recaptured | Full | SF15 | Successful |
|  | 2009/10 | Recaptured | Failed |  | Successful |
